# Poor performance of predictive equations to estimate resting energy expenditure in patients with Crohn’s disease

**DOI:** 10.1017/S000711452200068X

**Published:** 2023-01-28

**Authors:** Alexandra Karachaliou, Costas Anastasiou, Maria Bletsa, Gerassimos J. Mantzaris, Emmanuel Archavlis, George Karampekos, Maria Tzouvala, Eirini Zacharopoulou, Chrysoula Veimou, Giorgos Bamias, Meropi Kontogianni

**Affiliations:** 1 Department of Nutrition and Dietetics, School of Health Sciences and Education, Harokopio University, 70 El. Venizelou Ave, 17671, Kallithea, Greece; 2 Department of Nutrition and Dietetics, ‘Sotiria’ Thoracic Diseases Hospital, 152 Mesogion Ave, 11527, Athens, Greece; 3 Department of Gastroenterology, ‘Evangelismos-Ophthalmiatreion Athinon-Polykliniki’ General Hospital, 45-47 Ypsilantou Str., 106 76, Athens, Greece; 4 Department of Gastroenterology, General Hospital of Nikaia Piraeus ‘Agios Panteleimon’-General Hospital Dytikis Attikis ‘Agia Varvara’, 3 Dim. Mantouvalou Str., 184 54, Athens, Greece; 5 GI-Unit, 3rd Academic Department of Internal Medicine, ‘Sotiria’ Thoracic Diseases Hospital, Medical School, National and Kapodistrian University of Athens, 152 Mesogion Ave, 115 27, Athens, Greece

**Keywords:** Crohn’s disease, Resting energy expenditure, Accuracy, Predictive equations, Indirect calorimetry

## Abstract

Studies exploring the accuracy of equations calculating resting energy expenditure (REE) in patients with Crohn’s disease (CD) are lacking. The aim of this study was to investigate the accuracy of REE predictive equations against indirect calorimetry in CD patients. REE was measured using indirect calorimetry (mREE) after an overnight fasting. Fourteen predictive equations, with and without body composition analysis parameters, were compared with mREE using different body weight approaches. Body composition analysis was performed using dual X-ray absorptiometry. One hundred and eighty-six CD outpatients (102 males) with mean age 41·3 (sd 14·1) years and 37·6 % with active disease were evaluated. Mean mREE in the total sample was 7255 (sd 1854) kJ/day. All equations underpredicted REE and showed moderate correlations with mREE (Pearson’s r or Spearman’s rho 0·600–0·680 for current weight, all *P*-values < 0·001). Accuracy was low for all equations at the individual level (28–42 and 25–40 % for current and adjusted body weight, respectively, 19–33 % for equations including body composition parameters). At the group level, accuracy showed wide limits of agreement and proportional biases. Accuracy remained low when sample was studied according to disease activity, sex, BMI and medication use. All predictive equations underestimated REE and showed low accuracy. Indirect calorimetry remains the best method for estimating REE of patients with CD.

Resting energy expenditure (REE) is the amount of energy required for maintaining normal body functions, such as cardiovascular, brain and cell function, over a 24-h period at rest^(1)^. REE is estimated either via direct or indirect calorimetry or using available predictive equations^(2,3)^. In the clinical practice, REE is widely predicted based on several equations that are available in the literature^(4)^.

Crohn’s disease (CD) is an inflammatory bowel disease characterised by alterations of REE mainly due to the presence of active inflammation^(2)^. Patients with active disease usually experience an increase in their REE compared with patients in remission^(2,3)^. However, inconsistent data were observed among available studies regarding REE and disease activity^(4)^. Possible explanations for this might be an altered body composition due to inflammation (mostly a decrease in fat-free mass (FFM) and an increase in fat mass), drugs (especially corticosteroids) and lower levels of physical activity especially during the acute phases of the disease^(4–7)^. Studies exploring the accuracy of several predictive equations in patients with CD are scarce and rather old with only three studies examining the use of Harris–Benedict equations in patients with CD^(8–10)^. According to their findings, predicted REE (pREE) did not differ from measured REE (mREE) irrespectively from disease activity. Based on these limited results, the European Society for Clinical Nutrition and Metabolism (ESPEN) guidelines, published in 2017, mentions that predictive equations are suitable for these patients, since no consistent associations have been found between REE and disease activity and indirect calorimetry should be used in troublesome cases^(4)^.

However, the abovementioned studies exhibit several methodological limitations such as small sample sizes and different ways of REE estimation^(8–10)^, whereas they have used current body weight for the calculation of REE and there are not available studies examining whether use of adjusted body weight for obese patients could improve accuracy of REE. In addition, the accuracy of predictive equations including body composition parameters has not been yet explored. Acknowledging some of these limitations, Marra *et al*.^(11)^ recently examined the accuracy of several predictive equations in patients with CD and found that most of them underestimate REE leading them to the development of new disease-specific equations for this population. However, the reliability of these equations has not been explored yet^(11)^.

Recognising the limitations of utility of indirect calorimetry in clinical settings and the need for accurate prediction of REE in patients with CD, the aim of the present study was to investigate the accuracy of widely used REE predictive equations in patients with CD against REE measurement through indirect calorimetry. Additional exploratory aim was to estimate the accuracy of these equations according to disease activity, sex, medication use, BMI categories and body weight used in the predictive equations.

## Materials and methods

### Study sample

The study sample consisted of outpatients with CD participating in a cross-sectional nutritional status evaluation study. Inclusion criteria included patients > 16 years, with confirmed CD for more than 6 months and without previous history of cancer. All patients were enrolled from three outpatient gastroenterology clinics (Department of Gastroenterology, ‘Evangelismos-Ophthalmiatreio Athinon-Polykliniki’ General Hospital of Athens, Department of Gastroenterology, General Hospital of Nikaia Piraeus ‘Agios Panteleimon’-General Hospital Dytikis Attikis ‘Agia Varvara’ and GI-Unit, 3rd Academic Department of Internal Medicine, ‘Sotiria’ Hospital of Athens) from November 2018 to November 2019. Diagnosis was based on biopsy and a combination of clinical, histological, endoscopical, imaging and biochemical data. Exclusion criteria included hospitalised patients, patients with CD for less than 6 months, < 16 years old, with short bowel syndrome, cancer or history of cancer, chronic heart or kidney disease, nutrition support (parenteral nutrition) and for females, a period of gestation and lactation. For the current analyses, further exclusion criteria included not following instructions for REE measurement (i.e. overnight fasting for 12 h, avoid physical activity for 48 h and smoking the day of the test) and patients following a specific weight loss diet for more than 2 weeks.

### Ethical approval

The study was conducted according to the guidelines laid down in the Declaration of Helsinki, and all procedures involving human patients were approved by the Bioethics Committee of Harokopio University, the Scientific Committees of the three participating hospitals and were registered in ClinicalTrials.gov (NCT03871634). Written informed consent was obtained from all patients.

### Medical assessment

Data regarding basic socio-demographic characteristics (education level, marital status), smoking status, co-morbidities and medical treatment were recorded from collaborating physicians during medical assessment. Montreal classification was used for the categorisation of patients according to age at diagnosis (< 16, 17–40, > 40 years), disease location (ileal, colonic, ileocolonic) and behaviour (non-structuring/non-penetrating, structuring, penetrating)^(12)^. Clinical manifestations, such as abdominal pain, diarrhoea, blood loss, fever, weight loss and extra-intestinal manifestations, as well as their severity were also recorded. Disease activity was evaluated by endoscopy and using the Harvey–Bradshaw Index (HBI)^(13)^. Patients with HBI < 5 and normal C-reactive protein levels (< 5 mg/l)^(14)^ were categorised as in remission.

### Anthropometry and body composition

Body weight was measured to the nearest 0·1 kg and body height to the nearest 0·1 cm using a Seca 770 analogue body scale and stadiometer (Seca Alpha, Model 770). Subjects were instructed to wear light clothes and were measured without shoes. BMI was calculated dividing weight (in kg) by the square of height (in meters)^(15)^. Body composition analysis was performed using the dual X-ray absorptiometry method (Lunar DPX-MD; Lunar Corp.).

### Resting energy expenditure

REE was measured via indirect calorimetry using a mouthpiece and nose-clip system (Ultima™ Series PFX® with GC, MedGraphics Cardiorespiratory Diagnostics Corporation) for about 20–30 min. Calibration was conducted before each measurement using a gas mixture with known concentrations of oxygen analyser and carbon dioxide analyser (gas 1: CO_2_:4 %, O_2_:16 %, gas 2: CO_2_:0 %, O_2_:26 %). Before REE measurement, subjects were instructed to follow an overnight fasting (only water permitted), avoid physical activity for 48 h and not smoke the day of the test. REE measurement was conducted in a quiet, temperature and humidity-controlled environment, with the subject sitting in a slight incline position for 15 min before the test. REE measurement was finished when the subject was in a steady condition at least for 5 min, as indicated by RQ, oxygen consumption and minute ventilation rate. REE was calculated using the Weir formula^(16)^, without using protein oxidation^(17)^.

#### Predictive equations

mREE from indirect calorimetry was compared with pREE from fourteen widely used predictive equations for general population. Harris–Benedict^(17)^, Schofield^(18)^, FAO/WHO/UNU^(19)^, Mifflin^(20)^, Owen^(21,22)^, Muller^(23)^ and Marra equations, specific for CD patients^(11)^ were tested (online Supplementary Table S1). Also, Mifflin-St. Jeor^(20)^, Owen^(21,22)^, Muller^(23)^, Huang^(24)^, Wang^(25)^, Cunningham^(26)^ and Johnstone^(27)^ equations with a measure of body composition (FFM and/or fat mass) were also included (online Supplementary Table S1). For the calculations of REE from predictive equations, three different approaches for body weight were used. The first approach used current body weight for all patients with CD. The second approach used current body weight for all patients with CD except for obese patients (BMI ≥ 30 kg/m^2^) for whom an adjusted body weight as ((current body weight + ideal body weight)/2) was used^(28)^. The third approach used current body weight for all patients with CD except for obese patients (BMI ≥ 30 kg/m^2^) for whom another formula for adjusted body weight was used, namely ((current-ideal)/4 + ideal body weight)^(29)^. Ideal body weight was calculated as the body weight corresponding to BMI 25 kg/m^2^ for both equations for adjusted body weight^(28,29)^. Adjusted body weight was used for patients with obesity, since it accounts for the increased proportion of body weight as low metabolically active adipose tissue in obese people^(30)^. Initially, results from each predictive equation were not multiplied with a stress factor neither for active disease nor for remission.

### Statistical analysis

For the needs of the current analysis, based on an error limit of prediction accuracy of 10 % between mREE and pREE^(17,31)^ a total of ninety-five patients would be required to achieve a 90 % statistical power at a 5 % significance level (i.e. 95 % CI).

Statistical analysis was performed using IBM SPSS version 23 (IBM Corp. Released 2016. IBM SPSS Statistics for Windows, Version 24·0. IBM Corp.) and STATA version 15 (M. Psarros & Assoc.). The statistical significance threshold was set at 0·05. The normality of continuous variables was evaluated with Shapiro–Wilk test and graphically with Q-Q plots. Categorical variables were presented as relative and absolute frequencies. Data are presented as mean values and standard deviations for normally distributed variables and as median and 25th and 75th percentiles for non-normally distributed variables. Differences between active disease and remission were evaluated using independent *t* test or Mann–Whitney rank tests for normally and non-normally distributed continuous variables, respectively, and *χ* ^2^ tests for categorical variables. Differences between BMI categories were evaluated using one-way ANOVA (for normally distributed continuous variables) and Kruskal–Wallis test (for non-normally distributed continuous variables). Tukey’s test or multiple Mann–Whitney rank tests were used at *post hoc* analyses for the recognition of groups which differed significantly.

Pearson’s and Spearman’s correlation coefficients were used to test associations between mREE and pREE for normally distributed and skewed variables, accordingly. For each predictive equation, linear regression analysis was performed for the calculation of root mean square error (RMSE). Bland–Altman analysis was performed for the assessment of agreement between data from indirect calorimetry and data from each predictive equation and the estimation of the corresponding limits of agreement. Bias was calculated as average difference between pREE minus mREE, with positive values representing overpredictions of mREE and negative values underpredictions of mREE. Also, bias has been presented as a percentage of mREE. The corresponding limits of agreement were defined as bias ± 1·96 × sd. Subjects outside limits of agreement refer to patients whose bias was over 1·96 × sd or under −1·96 × sd in Bland–Altman analyses. Bias within this range is considered as acceptable^(32)^, whereas bias outside this range is considered as large, meaning that the equation cannot accurately predict REE in the group level. Pearson’s or Spearman’s correlation coefficients as a measure of agreement were also calculated. A percentage of patients with CD whose pREE was within ±10 % of mREE was considered as a measure of accuracy at an individual level^(31)^. At the group level, Bland–Altman method was used and plots presented the difference between pREE and mREE in the vertical axis and the mean of pREE and mREE in the horizontal axis^(32)^.

Pearson’s and Spearman’s correlation coefficients were used to test associations between the variation of pREE and several predictors (i.e. BMI, weight, age, CRP, HBI, sex, fat mass and FFM) for normally distributed and skewed variables, accordingly. Variables being significantly correlated with the variation of pREE at a significance level < 0·1 were then included in multivariate linear regression models.

## Results

**Fig. 1.**
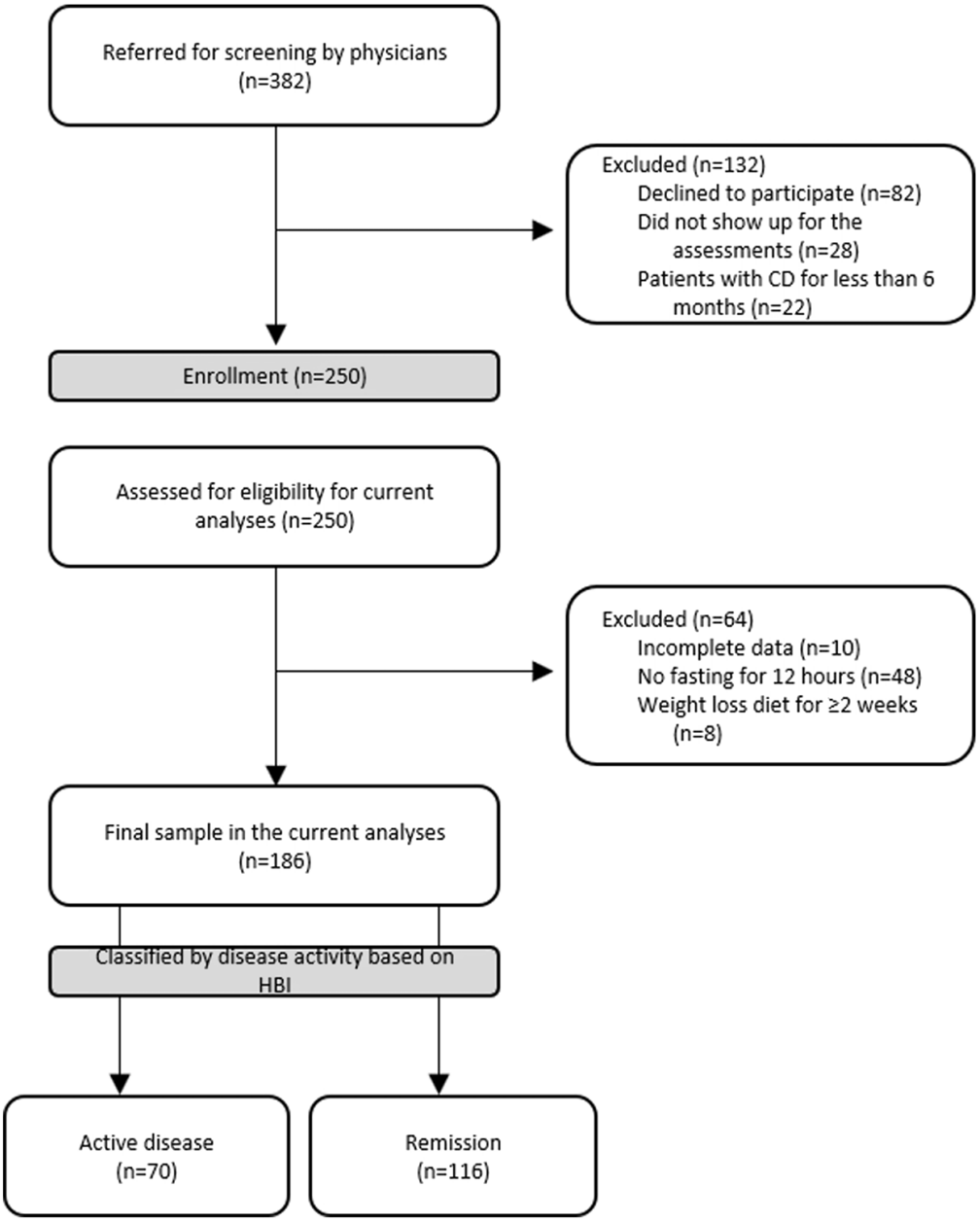
CONSORT chart. From November 2018 to November 2019, 382 patients with CD were referred by collaborating physicians for screening. Of the 382 patients, eighty-two declined to participate, twenty-eight did not show up for the assessments and twenty-two were diagnosed with CD for less than 6 months. In total, 250 patients with CD were included in a cross-sectional evaluation of nutritional status and assessed for eligibility in the current analyses. Ten patients who did not have complete data, eight patients who followed a weight loss diet for more than 2 weeks and forty-six patients who did not follow instructions for REE measurement (i.e. overnight fasting, avoid physical activity for 48 h and smoking the day of the test) were excluded from the present analyses, leading to a final sample of 186 patients. These patients were further classified by disease activity based on HBI to seventy patients with active CD and 116 patients in remission. CD, Crohn’s disease; HBI, Harvey–Bradshaw Index; REE, resting energy expenditure.

In total, 250 patients with CD included in a cross-sectional evaluation of nutritional status were assessed for eligibility in the current analyses. Ten patients who did not have complete data, eight patients who followed a weight loss diet for more than 2 weeks and forty-six patients who did not follow instructions for REE measurement (i.e. overnight fasting, avoid physical activity for 48 h and smoking the day of the test) were excluded from the present analyses, leading to a final sample of 186 patients (Fig. 1) (102 males, mean age 41·3 (sd 14·1) years and mean BMI 27·1 (sd 5·9) kg/m^2^). Most of the patients were diagnosed between 17 and 40 years old (65·6 %), had ileal (48·4 %) or ileocolonic disease (41·3 %) and a small percentage of them (10·3 %) had isolated upper gastrointestinal disease. Based on BMI categories, 3·2 % of patients were underweight (BMI < 18·5 kg/m^2^), 32·3 % were overweight (BMI 25–29·9 kg/m^2^) and 27·4 % were obese (BMI ≥ 30 kg/m^2^). Patients’ characteristics according to disease activity are presented in Table 1. Regarding medication, patients with active disease reported more frequent use of corticosteroids, whereas the majority of patients in remission received 5-aminosalicylic acid, azathioprine and biologic agents (data not shown).

**Table 1.** Descriptive characteristics of 186 patients with Crohn’s disease according to disease activity (Mean values and standard deviations)

	Total sample (*n* 186)†		Active disease (*n* 70)†		Remission (*n* 116)†		*P* ‡
	Mean	sd	Mean	sd	Mean	sd	
Age (years)	41·3	14·1	41·9	15·0	40·9	13·6	0·65
Sex	*n*	(%)	*n*	(%)	*n*	(%)	0·09
Male	102	54·8	44	62·9	58	50·0	
Education level	*n*	(%)	*n*	(%)	*n*	(%)	0·44
<6 years	16	8·6	7	10·0	9	7·8	
6–12 years	76	41·1	32	45·7	44	38·3	
>12 years	93	50·3	31	44·3	62	53·9	
Marital status	*n*	(%)	*n*	(%)	*n*	(%)	0·08
Unmarried	81	43·5	31	44·3	50	43·1	
Married	93	50·0	31	44·3	62	53·4	
Divorced/Widowed	12	6·5	8	11·4	4	3·4	
Smoking status	*n*	(%)	*n*	(%)	*n*	(%)	0·56
Smokers	85	45·9	36	51·4	49	42·6	
Body weight (kg)	77·5	18·4	79·9	20·1	76·0	17·2	0·18
Height (m)	1·69	0·10	1·69	0·10	1·69	0·10	0·79
BMI (kg/m^2^)	27·1	5·9	27·9	6·7	26·6	5·4	0·16
BMI categories	*n*	(%)	*n*	(%)	*n*	(%)	0·25
<18·5 kg/m^2^	6	3,2	1	1·4	5	4·4	
18·5–24·9 kg/m^2^	69	37·1	28	40·0	41	35·3	
25·0–29·9 kg/m^2^	60	32·3	18	25·7	42	36·2	
≥30·0 kg/m^2^	51	27·4	23	32·9	28	24·1	
FFM (kg)*	51·8	11·6	52·0	11·2	51·6	11·9	0·47
FM (kg)*	24·3	11·9	25·4	13·2	23·8	11·2	0·46
Disease duration (years)							
Median	7·0		6·0		8·0		0·58
25th–75th percentiles	3·0–13·8		1·0–14·0		3·0–13·0		
Age at diagnosis	*n*	(%)	*n*	(%)	*n*	(%)	0·07
<16 years (A1)	13	7·0	1	1·4	12	10·3	
17–40 years (A2)	122	65·6	49	70·0	73	62·9	
>40 years (A3)	51	27·4	20	28·6	31	26·7	
Disease location	*n*	(%)	*n*	(%)	*n*	(%)	0·04
Ileal (L1)	89	48·4	36	52·2	53	46·1	
Colonic (L2)	19	10·3	2	2·9	17	14·8	
Ileocolonic (L3)	76	41·3	31	44·9	45	39·1	
Isolated upper GI disease (L4)	21	11·3	11	15·7	10	8·6	0·14
Disease behaviour	*n*	(%)	*n*	(%)	*n*	(%)	0·47
Non-structuring, non-penetrating (B1)	112	60·2	40	57·1	72	62·1	
Structuring (B2)	49	26·3	22	31·4	27	23·3	
Penetrating (B3)	16	8·6	4	5·7	12	10·3	
B2 + B3	9	4·8	4	5·7	5	4·3	
Perianal disease (P)	25	13·4	9	12·9	16	13·8	0·86
HBI							
Median	2·0		5·0		1·0		<0·001
25th–75th percentiles	1·0–4·0		2·0–6·0		0·0–2·0		
CRP (mg/l)							
Median	3·30		3·95		3·10		0·02
25th–75th percentiles	1·60, 8·20		2·40–11·00		1·10–6·00		

CRP, C-reactive protein; GI, gastrointestinal disease; FFM, fat-free mass; FM, fat mass; EBI, Harvey–Bradshaw Index.

* Fat-free mass and fat mass were measured using dual-energy X-ray absorptiometry.

† Data for continuous variables are presented as means (standard deviation) or median (and 25th–75th percentiles) and for categorical variables as absolute numbers and relative frequencies.

‡ *P*-values were calculated using independent *t* test or Mann–Whitney rank tests for normally and non-normally distributed continuous variables, respectively, and *χ* ^2^ tests for categorical variables.

### Comparisons of measured resting energy expenditure with predicted resting energy expenditure for equations without body composition analysis parameters

Mean mREE in the total sample was 7255 (sd 1854) kJ/d (range: 2941–13544 kJ/d) (Table 2). mREE was compared with pREE using different approaches for body weight. All calculations based on current body weight underpredicted REE. Harris–Benedict and Marra equations were closer to mREE, while Mifflin and Owen ones showed the lowest pREE from all equations (Table 2). Also, pREE was compared with mREE graphically showing that all equations underestimated mREE (online Supplementary Fig. S1).

**Table 2.** Evaluation of REE from predictive equations without stress factor in 186 patients with Crohn’s disease (Mean values and standard deviations; median values and percentiles)

	REE kJ/d‡		Pearson’s r or Spearman’s rho	RMSE kJ/d	Accurate predictions (% subjects)†	Under predictions (% subjects)	Over predictions (% subjects)
	Median	25th–75th percentiles					
Measured							
Mean	7255						
sd	1854						
Predictive equations without body composition parameters							
Schofield with current BW							
Mean	6937		0·638*	22·6	39	37	24
sd	1209						
Schofield with adjusted BW§	6761	5849–7581	0·636*	41·4	34	44	22
FAO/WHO/UNU with current BW	6832	5929–7711	0·634*	22·2	39	38	23
FAO/WHO/UNU with adjusted BW§	6711	5812–7581	0·632*	40·6	34	45	21
Harris–Benedict with current BW	7104	6159–8100	0·655*	4·2	42	30	28
Harris–Benedict with adjusted BW§	6845	6130–7786	0·657*	18·8	40	35	25
Mifflin with current BW							
Mean	6506		0·680*	45·2	31	54	15
sd	1172						
Mifflin with adjusted BW§							
Mean	6351		0·677*	67·8	25	60	15
sd	1084						
Owen with current BW	6473	5414–7247	0·600*	65·7	28	55	17
Owen with adjusted BW§	6305	5376–7150	0·596*	82·0	25	60	15
Muller with current BW							
Mean	6773		0·619*	27·6	39	42	19
sd	1197						
Muller with adjusted BW§	6648	5702–7439	0·616*	54·8	31	52	17
Marra with current BW							
Mean	7075		0·659*	8·4	42	32	26
sd	1105						
Marra with adjusted BW§							
Mean	6907		0·671*	15·9	39	38	23
sd	979						
Predictive equations with body composition parameters							
Mifflin-St. Jeor	5979	5138–6774	0·607*	115·1	21	67	12
Owen	5925	4824–6899	0·598*	139·3	19	69	12
Muller	6702	5669–7364	0·615*	40·2	36	45	19
Huang	6485	5376–7330	0·610*	59·0	31	53	16
Wang	6343	5427–7209	0·607*	94·1	30	54	16
Cunningham	6209	5289–7079	0·607*	104·2	25	59	16
Johnstone	6489	5665–7372	0·625*	47·7	33	48	19

BW, body weight; REE, resting energy expenditure; RMSE, root mean square prediction error.

* *P*-values < 0·001.

† Predicted REE is considered accurate if it is within ±10 % of measured REE.

‡ Data are presented as means (standard deviation) or median (and 25th-75th percentiles).

§ Adjusted BW: (current BW + ideal BW)/2 for obese patients (BMI ≥ 30 kg/m^2^), current BW for underweight, normal weight and overweight patients. Ideal BW is corresponded to BMI 25 kg/m^2^.

When adjusted body weight for patients with BMI ≥ 30 kg/m^2^ was used as ((current body weight + ideal body weight)/2), predictive equations underestimated REE and difference between mREE and pREE was higher, indicating that current body weight was a better choice than adjusted body weight for calculating energy expenditure in obese patients with CD (Table 2). Calculations based on adjusted body weight for patients with BMI ≥ 30 kg/m^2^ estimated as ((current body weight – ideal body weight)/4 + ideal body weight) presented even larger underestimation of mREE and lower accuracy compared with other approaches for body weight and were no further explored (data not shown).

### Accuracy at the individual level

**Fig. 2.**
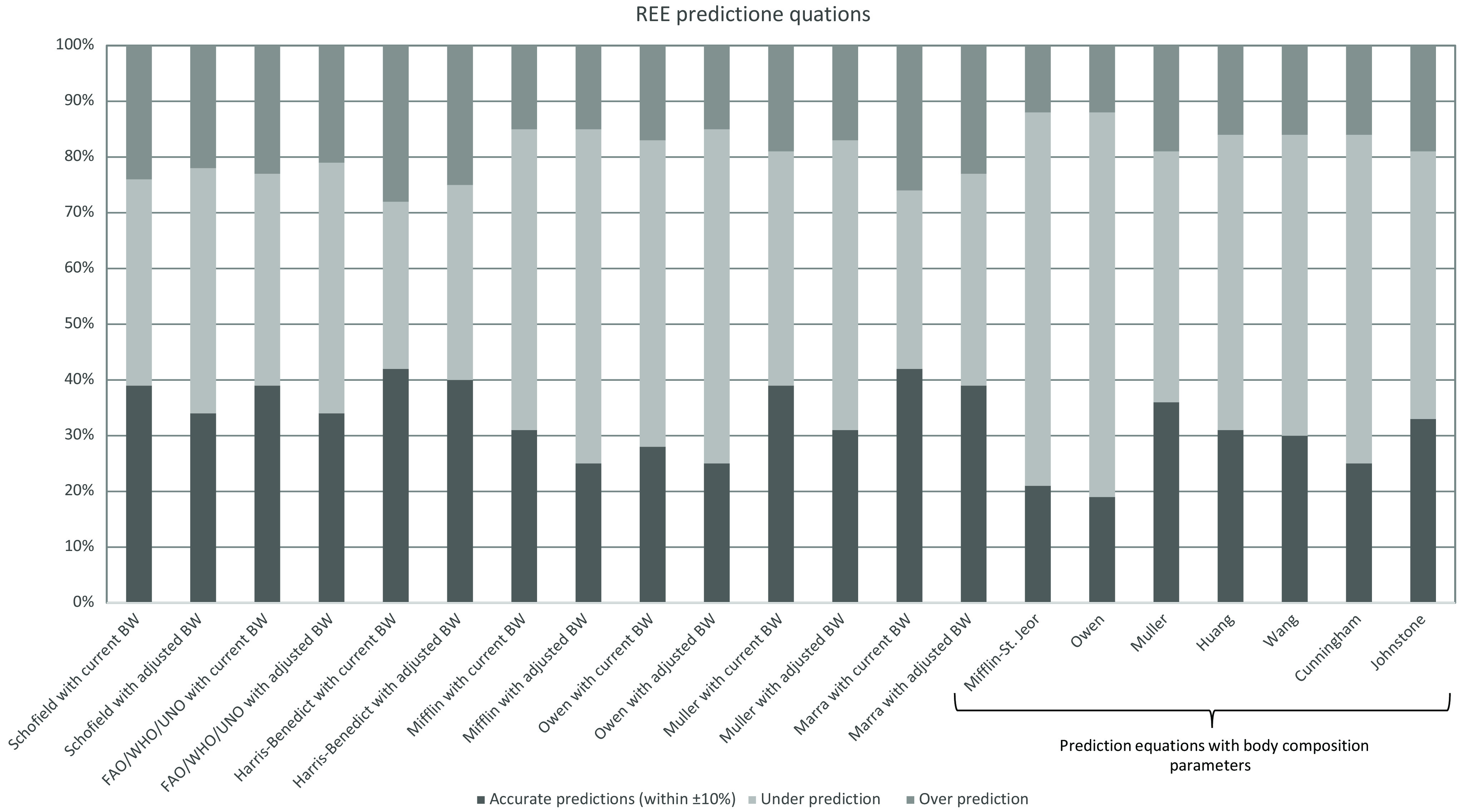
Proportion of predictive REE equations within ±10 % of measured REE. A predictive REE equation was considered accurate if it was within ±10 % of measured REE. If accuracy was < 90 %, the predictive equation underestimated REE. If accuracy was > 110 %, the predictive equation overestimated REE. Adjusted BW: (current BW + ideal BW)/2 for obese patients (BMI ≥ 30 kg/m^2^), current BW for underweight, normal weight and overweight patients. Ideal BW is corresponded to BMI 25 kg/m^2^. BW, body weight; REE, resting energy expenditure.

When accuracy of pREE at the individual level was assessed, all predictive equations without body composition parameters showed similar results regarding correlations with mREE (Pearson’s r or Spearman’s rho 0·600–0·680 for current weight and 0·596–0·677 for adjusted weight, all *P*-values < 0·001, Table 2). Moderate correlations were observed when pREE was calculated with equations including body composition parameters (Pearson’s r or Spearman’s rho 0·598–0·625, all *P*-values < 0·001). Owen equations without body composition variables showed the worst results with less strong correlations and higher RMSE, while Harris–Benedict and Marra equations showed the best results and lower RMSE. The proportion of pREE equations within ±10 % of mREE is presented in Fig. 2. Harris–Benedict and Marra equations with current body weight showed the highest accuracy within ±10 % of mREE (42 and 41 %, respectively) (Fig. 2). However, all predictive equations without body composition analysis parameters showed low accuracy within ±10 % of mREE. More than half of the patients’ REE were not accurately predicted with existing equations. In specific, accuracy was low for all equations at the individual level (28–42 % for current body weight and 25–40 % for adjusted body weight) (Table 2).

### Accuracy at the group level

pREE accuracy was further explored at the group level by performing the Bland–Altman analyses (Table 3, online Supplementary Fig. S2). Bias (as a percentage and as kJ/d), 95 % CI for the bias, limits of agreement and subjects outside limits of agreement are presented in Table 3. Schofield equations with current body weight showed the best accuracy at the group level (bias −0·5 %, limits of agreement −3125, 2494 kJ) followed by FAO/WHO/UNU (bias −0·97 %, limits of agreement −3163, 2473 kJ) and Marra equations, which showed the smallest limits of agreement among all equations (bias 1·69 %, limits of agreement −2929, 2565 kJ, respectively). However, correlations between difference of each pREE and mREE with mean REE were moderate (rho 0·438–0·578, all *P*-values < 0·001).

**Table 3.** Bland–Altman analyses for the agreement between measured REE from indirect calorimetry and REE from predictive equations in 186 patients with Crohn’s disease (Mean values and standard deviations)

	Bias (kJ/d)†		Bias (%)†		95 % CI for the bias (kJ/d)	Limits of agreement (mean ± 1.96 sd) (kJ/d)		Absolute limits of agreement (kJ/d)	Subjects outside limits of agreement (%)	Rho*
	Mean	sd	Mean	sd		Lower	Upper			
Predictive equations without body composition parameters										
Schofield with current BW	–318	1435	–0·50	22·0	–523, −109	–3125	2494	5619	4·3	–0·464*
Schofield with adjusted BW‡	–485	1427	–2·71	21·0	–690, −276	–3280	2314	5594	4·3	–0·576*
FAO/WHO/UNU with current BW	–343	1439	–0·97	21·8	–556, 138	–3163	2473	5636	4·3	–0·461*
FAO/WHO/UNU with adjusted BW‡	–510	1431	–3·16	20·9	–715, −305	–3314	2293	5607	4·3	–0·567*
Harris–Benedict with current BW	–42	1397	–3·32	22·7	–243, 159	–2782	2699	5481	4·8	–0·438*
Harris–Benedict with adjusted BW‡	–230	1381	0·82	21·4	–427, −29·7	–2933	2473	5406	3·8	–0·562*
Mifflin with current BW	–749	1364	–7·0	19·6	–946, −552	–3423	1925	5347	4·3	–0·533*
Mifflin with adjusted BW‡	–904	1377	–9·08	18·8	–1100, −703	–3602	1799	5402	4·3	–0·598*
Owen with current BW	–833	1485	–7·7	21·4	–1046, −613	–3740	2075	5816	4·3	–0·528*
Owen with adjusted BW‡	–966	1490	–9·44	20·6	–1184, −749	–3887	1929	5841	4·3	–0·591*
Muller with current BW	–481	1443	–2·9	21·9	–690, −272	–3310	2343	5653	4·3	–0·487*
Muller with adjusted BW‡	–665	1439	–5·37	20·9	–874, −460	–2820	2151	4971	4·3	–0·584*
Marra with current BW	–180	1402	1·69	22·1	–385, 21·8	–2929	2565	5494	4·8	–0·578*
Marra with adjusted BW‡	–347	1402	–0·54	21·2	–548, −146	–3096	2402	5498	4·3	–0·655*
Predictive equations with body composition parameters										
Mifflin-St. Jeor	–1201	1448	–13·1	19·1	–1414, −992	–4038	1636	5674	4·4	–0·622*
Owen	–1280	1456	–14·8	19·4	–1490, −1067	–4142	1586	5728	3·8	–0·448*
Muller	–569	1439	–3·95	21·4	–782, −364	–3397	2255	5653	4·9	–0·580*
Huang	–766	1439	–7·11	20·9	–971, −552	–3586	2059	5644	4·4	–0·473*
Wang	–837	1439	–7·93	20·2	–1046, −628	–3656	1983	5640	4·4	–0·563*
Cunningham	–971	1439	–9·92	19·8	–1180, −761	–3791	1849	5640	4·4	–0·560*
Johnstone	–649	1406	–5·48	20·7	–854, −444	–3406	2109	5515	4·4	–0·502*

BW, body weight; REE, resting energy expenditure.

* Spearman’s correlation coefficients between mean and difference REE from each predictive equation and REE from indirect calorimetry. All *P*-values < 0·001.

† Bias is defined as the mean difference between measured REE and predicted REE.

‡ Adjusted BW: (current BW + ideal BW)/2 for obese patients (BMI ≥ 30 kg/m^2^), current BW for underweight, normal weight and overweight patients. Ideal BW is corresponded to BMI 25 kg/m^2^.

Regarding adjusted body weight, Harris–Benedict equations showed the best accuracy at the group level (bias 0·82 %, limits of agreement −2933, 2473 kJ). Moderate positive correlations remained in all equations (rho = 0·562–0·655, all *P*-values < 0·001). Mifflin and Owen equations showed the worst results both for current and adjusted body weight at the group level (Table 3).

### Comparisons of measured resting energy expenditure with predicted resting energy expenditure and accuracy at the individual and group level for equations with body composition analysis parameters

Except for equations without body composition parameters, pREE was also calculated using equations with body composition analysis parameters. All these equations yielded REE values lower than mREE and even lower than pREE from equations without body composition analysis (Table 2). Muller equations showed more closer results to mREE with lower RMSE, while Owen equations showed the worst results compared with the rest of equations based on body composition analysis parameters (Table 2).

Moreover, accuracy of these equations has been assessed at the individual level. Owen equations again showed the worst results with less strong correlations and higher RMSE, while Harris–Benedict and Marra equations showed the best results (Table 2). However, all predictive equations with body composition analysis parameters showed lower accuracy within ±10 % of mREE compared with those without body composition analysis parameters (19–33 %) (Table 2, Fig. 2).

When accuracy was assessed at the group level, all equations showed wider limits of agreement and worse results than equations without body composition parameters (Table 3, online Supplementary Fig. S2). It should be noted that all predictive equations used in the current study showed wide limits of agreement and proportional biases with the difference between pREE and mREE getting larger as mean REE was increasing.

### Comparison between measured resting energy expenditure and predicted resting energy expenditure in terms of disease activity

Further sensitivity analyses were conducted to investigate whether accuracy of the predictive equations differed depending on disease activity. Mean mREE was compared with mean pREE from different equations used in the current study within and between active disease and remission. Within these groups, all predictive equations underestimated REE and showed moderate correlations with mREE (all *P*-values < 0·001, data not shown). Between groups, pREE and mREE did not differ (all *P*-values > 0·05). Worse results were observed when adjusted body weight for obese patients was used for calculations of REE predictive equations (online Supplementary Table S2).

Moreover, accuracy of pREE was further investigated at the individual (online Supplementary Table S2) and group level (online Supplementary Table S3) according to the disease activity. Based on these results, accuracy remained low (17–40 % in active disease and 19–44 % in remission) at the individual level and presented wide limits of agreement and proportional biases (0·467–0·693 in active disease and 0·447–0·645 in remission, all *P*-values < 0·001) at the group level according to the Bland–Altman analyses. Worse results were observed when adjusted body weight for obese patients was used for calculations of REE from all predictive equations and from REE equations with body composition analysis parameters.

Since accuracy remained low when sample was studied according to disease activity groups, different stress factors between 0 and 10 % based on the Elia graph^(33)^ were applied in all predictive equations to explore any improvements of accuracy. All equations were adjusted for a stress factor either in active disease only or in active disease and remission. Although several stress factors were applied in all equations, the accuracy was not improved (data not shown).

### Comparison between measured resting energy expenditure and predicted resting energy expenditure in terms of BMI categories, sex and medication use

Further exploratory analyses were performed according to BMI categories. Patients were categorised either into four BMI categories (< 18·5, 18·5–24·9, 25–29·9 and ≥ 30 kg/m^2^) or into five BMI categories (< 18·5, 18·5–24·9, 25–26·9, 27–29·9 and ≥ 30 kg/m^2^) by further dividing overweight patients into two categories based on median BMI (BMI 27 kg/m^2^). For both approaches, all predictive equations used in the current study tended to underestimate REE in all BMI categories, except for BMI 27–29·9 kg/m^2^ for Harris–Benedict, Schofield and FAO/WHO/UNU equations, which presented a trend of overestimation (data not shown). Moreover, accuracy of pREE was further investigated at the individual level according to BMI categories and results were similar with those for disease activity for all equations (all *P*-values > 0·05, data not shown). At the group level, accuracy remained low, with large CI and wide limits of agreement and proportional biases for all equations tested and in all BMI categories (online Supplementary Fig. S3). Regarding sex, males had higher REE compared with females and all predictive equations underestimated REE both in males and females. Moreover, according to sensitivity analyses for sex, the accuracy of pREE was low both at the individual and group level, with large CI and wide limits of agreement, as well as proportional biases for all equations tested in either sex category (data not shown). Regarding medication, several analyses were performed according to medication categories (i.e. 5-ASA, azathioprine, methotrexate, corticosteroids, biologic agents); however, results remained practically unchanged not supporting that medication could be responsible for the large bias of the pREE (data not shown).

### Associations between variation of predicted resting energy expenditure and potential predictors

To further explore which parameters could have predominantly affected bias of pREE, correlations between the variation of pREE and several parameters (i.e. BMI, weight, age, CRP, HBI, sex, fat mass and FFM) were explored. BMI, CRP, HBI and sex did not correlate with the variation of pREE, whereas age, weight, fat mass and FFM did, as presented in Table 4. Those variables correlated with variation of pREE at a significance level < 0·1 were then included in multivariate models. According to the results, only age was positively correlated with variation of pREE for all equations with body composition analysis parameters and for most of those without body composition analysis parameters, except for Harris–Benedict, FAO/WHO/UNU and Mifflin with current body weight.

**Table 4.** Correlations of resting energy expenditure variation of pREE with age, weight, fat mass and fat-free mass

	Age (years)	Weight (kg)	Fat mass (kg)	Fat-free mass (kg)
Predictive equations without body composition parameters				
Schofield with current BW	0·184***	0·056	–0·062	0·142
Schofield with adjusted BW	0·168***	–0·090	–0·097	0·005
FAO/WHO/UNU with current BW	0·145***	0·046	–0·067	0·129
FAO/WHO/UNU with adjusted BW	0·125	–0·100	–0·100	–0·010
Harris–Benedict with current BW	0·105	0·061	–0·067	0·141
Harris–Benedict with adjusted BW	0·067	–0·095	–0·105	–0·005
Mifflin with current BW	0·093	0·053	–0·019	0·096
Mifflin with adjusted BW	0·053	–0·104	–0·045	–0·062
Owen with current BW	0·339*	0·010	–0·038	0·058
Owen with adjusted BW	0·311*	–0·102	–0·062	–0·047
Muller with current BW	0·214**	0·081	–0·069	0·182**
Muller with adjusted BW	0·189**	–0·071	–0·092	0·032
Marra with current BW	0·260*	0·016	–0·141	0·166***
Marra with adjusted BW	0·228***	–0·124	–0·167***	0·029
Predictive equations with body composition parameters				
Mifflin-St. Jeor	0·265*	–0·093	–0·013	–0·108
Owen	0·250*	–0·001	0·169***	–0·146***
Muller	0·230**	–0·003	–0·103	0·095
Huang	0·279*	0·109	0·035	0·126
Wang	0·264*	–0·079	0·006	–0·051
Cunningham	0·264*	–0·064	0·028	–0·106
Johnstone	0·210**	0·105	–0·001	0·151***

BW, body weight.

* *P*-value < 0·001.

** *P*-value < 0·01.

*** *P*-value < 0·05.

## Discussion

In the current study, the accuracy of fourteen widely used REE predictive equations was explored in CD outpatients. According to the results, all predictive equations showed low accuracy both at the individual and group level (wide limits of agreement and proportional biases) and variation of pREE was positively associated with age.

Few studies have explored the accuracy of predictive REE equations in patients with CD and most of them have explored the Harris–Benedict equations but with conflicting results, finding that these equations either accurately predicted mREE^(34)^ or underestimated REE^(8,9)^. However, these studies had small sample sizes (< 55 patients), different inclusion criteria and methodological limitations^(8,9,34)^. In line with our results, Marra *et al*.^(11)^ also found that several equations underestimated REE in 270 CD outpatients. Moreover, all equations used in the current study could predict REE for a small proportion of patients with CD (< 40 %) within ±10 %, whereas those with body composition analysis parameters could predict even fewer patients.

Marra *et al*.^(11)^ also developed new disease-specific equations for patients with CD. In the current study, we compared these equations with mREE; however, also these equations showed low accuracy (42 %) with large negative and positive error at the individual level and wide limits of agreement (5494 kJ), and proportional biases at the group level. Although these equations are CD specific and have been suggested as to accurately predict REE of patients with CD, this was not confirmed in our study. Comparing our study with that of Marra *et al*., the most striking difference seems to be the weight status with our sample having mean weight 77·5 (sd 18·4) kg and mean BMI 27·1 (sd 5·9) kg/m^2^, whereas the validation group in the study of Marra *et al*. had mean weight 63·8 (sd 12·7) kg and mean BMI 21·9 (sd 3·7) kg/m^2^. This difference in weight status could partly explain the different results regarding accuracy of these equations at the individual level.

Since FFM affects REE, the use of predictive equations with FFM has been suggested in the past as more accurate than using equations based only on weight and height. However, in the present study, these equations underestimated REE more than equations without body composition variables and showed lower accuracy rates compared with other equations. Low accuracy of these equations may be due to the fact that FFM is a heterogeneous part of the body composition that contains tissues and organs with different metabolic responses^(35)^. FFM explains a large percentage (up to 80 %) of REE^(36)^. In addition, accuracy of REE equations using body composition analysis parameters could also be affected by the techniques applied in the development and assessment of FFM. Most of the available REE equations have been developed using bioelectrical impedance analysis or skinfolds as methods of body composition assessment, and none has used the dual X-ray absorptiometry, the method applied in the current study.

Possible associations between variation of pREE and potential parameters that could have contributed to this bias were further explored. Age was the only variable associated with variation of pREE in multivariate models and was positively associated with it for all equations except those of Harris–Benedict, FAO/WHO/UNU and Mifflin with current body weight, suggesting that as age is increasing it is associated with REE overprediction. The decrease in REE with age is well known and could be partly attributed to decreases in FFM, in protein synthesis and norepinephrine secretion^(37,38)^.

Adjusted body weight has been suggested for patients with obesity, since adipose tissue is not as metabolically active as lean tissue, so using current body weight may lead to overestimation of REE^(39)^. Most of the available equations have been developed for healthy non-obese populations using current body weight and the use of adjusted body weight has been proposed as a way of correction of REE estimation for obese patients^(40)^, although it has been found that adjusted body weight often underestimates REE in obese individuals^(40,41)^. In the present study, adjusted body weight for CD patients with BMI ≥ 30 kg/m^2^ was also used; however, accuracy was not improved compared with the results with current body weight.

Since all equations tested could predict REE with accuracy < 42 %, different stress factors of 0–10 % based on Elia *et al*.^(33)^ were applied either in active disease or both in active disease and remission to explore whether accuracy could be improved. A stress factor was applied in remission, since patients with CD appear a chronic low-grade inflammation persisting in this disease activity group, resulting that patients often experience an increase in their REE not only in active disease but also in remission^(42)^. However, accuracy was not improved and still presented large CI, wide limits of agreement and proportional biases. This could be partly attributed to the fact that the level of inflammation is probably different for each patient and depends on several factors, such as disease duration, location and drug therapy^(42,43)^. Consequently, patients with CD constitute a heterogeneous population and a specific percentage for stress factor for all patients would not improve accuracy, since each patient may need or not a different stress factor based on general clinical and endoscopic evaluation.

Based on current findings, the problem of exact REE estimation remains, given that no equation seems to accurately predict energy needs of patients with CD. Based on the existing equations and data, in the clinical practice where indirect calorimetry is not always available, it is recommended to start dietary intervention using one of these predictive REE equations and reassess CD patient at regular intervals to adjust dietary intake according to actual needs.

The present study has both strengths and limitations. This is the first study examining the accuracy of different REE predictive equations with and without body composition analysis parameters using current and adjusted body weight (for equations without body composition analysis) in patients with CD and according to disease activity, sex, age groups and BMI categories. In addition, study sample included a wide range of patients with CD (i.e. active disease and in remission, different disease location), allowing results to be extracted for the whole range of patients with CD. Furthermore, mREE was assessed by the gold-standard method, namely indirect calorimetry, for at least 20–30 min ensuring that the patients were in a steady-state condition with appropriate conditions to perform the measurement. Regarding limitations, most of patients with CD were in remission and a smaller percentage (37·6 %) had active disease, whereas some sensitivity analyses performed in small subgroups (i.e. patients with BMI < 18·5 kg/m^2^ and patients using methotrexate) may have been underpowered.

### Conclusions

All predictive equations used in the present study underestimated REE and showed low ability to accurately estimate actual resting energy needs of CD patients both at the individual and group level. Indirect calorimetry remains the best method for estimating REE of patients with CD.

## Supplementary material

For supplementary material accompanying this paper visit https://doi.org/10.1017/S000711452200068X. click here to view supplementary material
