# Supplementary material for: Poor performance of predictive equations to estimate resting energy expenditure in patients with Crohn’s disease
Source: Br J Nutr. 2022 Mar 7;129(2):272–82. doi: 10.1017/S000711452200068X (PMC9870717; doi:10.1017/S000711452200068X)
Supplement: Supplementary file 1 [file S000711452200068Xsup001.docx]

**Supplementary material**

**Supplementary Table 1. Description of 14 predictive equations used in the current study.**

| **Reference** | **Number of subjects** | **Sample’s age** | **Sample’s characteristics (weight, height or BMI)** | **Predictive equations** |
| --- | --- | --- | --- | --- |
| **Predictive equations without body composition parameters** | | | | |
| Schofield | 4814 | Adults >18 years | Normal weight | M: age ≤30: 15.057*weight + 692.2  M: 30< age ≤60: 11.472*weight + 873.1  F: age ≤30: 14.818*weight + 486.6  F: 30< age ≤60: 8.126*weight + 845.6 |
| FAO/WHO/UNU | 11000 | All ages | Including overweight and obese | M: 10-18y: REE (kcal/day) = 17.686*weight + 658.2  M: 18-30y: REE (kcal/day) = 15.057*weight + 692.2  M: 30-60y: REE (kcal/day) = 11.472*weight + 873.1  M: ≥60y: REE (kcal/day) = 11.711*weight + 587.7  F: 10-18y: REE (kcal/day) = 13.384*weight + 692.6  F: 18-30y: REE (kcal/day) = 14.818*weight + 486.6  F: 30-60y: REE (kcal/day) = 8.126*weight + 845.6  F: ≥60y: REE (kcal/day) = 9.082*weight + 658.5 |
| Harris-Benedict | 239 (136M, 103F) | 21 – 70 years | Weight: 25 – 124.9kg  Height: 151 – 200cm | M: REE (kcal/day) = 66.4730 + 13.7516*weight + 5.0033*height(cm) - 6.7550*age  W: REE (kcal/day) = 655.0955 + 9.5634*weight + 1.8496*height(cm) - 4.6756*age |
| Mifflin-St. Jeor | 498 (251M, 247F) | 19 – 78 years | 17 - 42kg/m^2^ | REE (kcal/day) 9.99*weight + 6.25*height (cm) – 4.92*age + 166*sex – 161 |
| Owen | 104 (60M, 44F) | 18 – 82 years | 18 - 59kg/m^2^ | M: REE (kcal/day) = 879 + 10.2*weight  F: REE (kcal/day) = 795 + 7.18*weight |
| Muller* | 1046 (388M, 658F) | 44 ± 17 years (mean ± SD) | 27 ± 8kg/m^2^  (mean ± SD) | REE (MJ/day) = 0.047*weight + 1.009*sex – 0.01452*age + 3.21) |
| Marra | 270 (159M, 111F) | 18 – 65 years | 22.2±3.4kg/m^2^  Including overweight and obese | REE (kcal/d) = 10.8*Weight + 6.42*height(cm)− 1.85*age − 211 (+102 if male) |
| **Predictive equations with body composition parameters** | | | | |
| Mifflin-St. Jeor | 498 (251M, 247F) | 19 – 78 years | 17 - 42kg/m^2^  FFM was calculated using the sum of three skinfold thicknesses, the thigh, triceps, and suprailium for women and the thigh, chest, and abdomen for men. | REE (kcal/d) = 19.7*FFM + 413 |
| Owen | 104 (60M, 44F) | 18 – 82 years | 18 - 59kg/m^2^  FFM was calculated using the sum of three skinfold thicknesses, the thigh, triceps, and suprailium for women and the thigh, chest, and abdomen for men. | M: REE (kcal/d) = 22.3*FFM + 290  W: REE (kcal/d) = 19.7*FFM + 334 |
| Muller* | 1046 (388M, 658F) | 44 ± 17 years (mean ± SD) | 27 ± 8kg/m^2^  (mean ± SD)  FFM was calculated using bioelectrical impedance analysis. | REE (MJ/d) = 0.05192*FFM + 0.04036*FM + 0.869*sex – 0.01181*age + 2.992  BMI ≤ 18.5: REE (MJ/d) = 0.08961*FFM + 0.05662*FM + 0.667  BMI 18.5–25: REE (MJ/d) = 0.0455*FFM + 0.0278*FM + 0.879*sex – 0.01291*age + 3.634  BMI 25-30: REE (MJ/d) = 0.03776*FFM + 0.03013*FM + 0.93*sex − 0.01196*age + 3.928  BMI ≥ 30: REE (MJ/d) = 0.05685*FFM + 0.04022*FM + 0.808*sex − 0.01402*age + 2.818 |
| Huang | 1038 (279M, 759W) | Adults  >18 years | BMI ≥ 35 kg/m^2^  Body composition was measured using the bioelectrical impedance analysis method. | REE (kcal/d) = 14.118*FFM + 9.367*FM − 1.515*age + 220.863*sex + 521.995 |
| Wang | 15 different equations  Body composition was measured using different techniques other than dual x-ray absorptiometry. | | | REE (kcal/d) = 21.5*FFM + 407 |
| Cunningham | 8 different equations for lean and obese subjects  Body composition was measured using different techniques other than dual x-ray absorptiometry. | | | REE (kcal/d) = 21.6*FFM + 370 |
| Johnstone* | 150 (43Μ, 107F) | 21 – 64 years | 16.7–49.3 kg/m^2^  Body fat was measured by air displacement whole body plethysmography and FFM was calculated using the sum of three skinfold thicknesses. | REE (kJ/d) = 90.2*FFM + 31.6*FM − 12.2*age + 1613 |

Age in years, BMI: Body mass index, FFM: Fat-free mass in kilograms, FM: Fat mass in kilograms, REE: Resting energy expenditure in kcal/day, sex (F: females = 0, M: males = 1), weight in kilograms.

*REE was calculated as MJ/d or kJ/day and then converted to kcal/day.

**Supplementary Table 2. Evaluation of REE from predictive equations in 186 patients with Crohn’s disease according to disease activity**

|  | **Active disease**  **(N=70)** | | | | | **Remission**  **(N=116)** | | | | |
| --- | --- | --- | --- | --- | --- | --- | --- | --- | --- | --- |
|  | **REE**  **Kcal/day** | **RMSE**  **Kcal/day** | **Accurate predictions (% subjects)^*^** | **Under predictions (% subjects)** | **Over predictions (% subjects)** | **REE**  **Kcal/day** | **RMSE**  **Kcal/day** | **Accurate predictions (% subjects)^*^** | **Under predictions (% subjects)** | **Over predictions (% subjects)** |
| **Measured** | 1738 (445) |  |  |  |  | 1732 (445) |  |  |  |  |
| **Predictive equations without body composition parameters** | | | | | | | | | | |
| **Schofield with current BW** | 1702 (293) | 73.7 | 40 | 33 | 27 | 1632 (284) | 6.9 | 39 | 38 | 23 |
| **Schofield with adjusted BW** | 1652 (1458, 1840) | 73.7 | 32 | 44 | 24 | 1549 (1368, 1802) | 12.6 | 37 | 42 | 21 |
| **FAO/WHO/UNU with current BW** | 1693 (1458, 1854) | 73.7 | 40 | 34 | 26 | 1605 (1371, 1836) | 6.7 | 39 | 39 | 22 |
| **FAO/WHO/UNU with adjusted BW** | 1642 (1425, 1840) | 73.7 | 33 | 44 | 23 | 1545 (1364, 1802) | 12.3 | 36 | 44 | 20 |
| **Harris-Benedict with current BW** | 1730 (1505, 1934) | 56.4 | 43 | 29 | 28 | 1663 (1458, 1935) | 1.3 | 41 | 30 | 29 |
| **Harris-Benedict with adjusted BW** | 1665 (1475, 1875) | 56.6 | 37 | 37 | 26 | 1600 (1455, 1851) | 5.7 | 42 | 33 | 25 |
| **Mifflin with current BW** | 1592 (289) | 78.5 | 31 | 50 | 19 | 1533 (274) | 13.7 | 30 | 56 | 14 |
| **Mifflin with adjusted BW** | 1542 (258) | 78.5 | 24 | 57 | 19 | 1504 (260) | 20.6 | 25 | 62 | 13 |
| **Owen with current BW** | 1576 (1396, 1740) | 86.3 | 29 | 51 | 20 | 1486 (1267, 1716) | 20.0 | 27 | 57 | 16 |
| **Owen with adjusted BW** | 1570 (1342, 1726) | 86.3 | 24 | 57 | 19 | 1430 (1267, 1704) | 25.0 | 25 | 61 | 14 |
| **Muller with current BW** | 1666 (292) | 76.2 | 40 | 40 | 20 | 1591 (280) | 8.4 | 38 | 43 | 19 |
| **Muller with adjusted BW** | 1623 (1394, 1781) | 76.2 | 31 | 50 | 19 | 1542 (1353, 1771) | 16.6 | 31 | 52 | 17 |
| **Marra with current BW** | 1726 (274) | 66.1 | 40 | 31 | 29 | 1670 (256) | 2.6 | 44 | 31 | 25 |
| **Marra with adjusted BW** | 1673 (233) | 66.1 | 36 | 40 | 24 | 1638 (236) | 4.9 | 42 | 36 | 22 |
| **Predictive equations with body composition parameters** | | | | | | | | | | |
| **Mifflin-St. Jeor (kcal/day)** | 1429 (1228, 1619) | 100 | 21 | 63 | 16 | 1412 (1223, 1621) | 34.5 | 21 | 71 | 8 |
| **Owen (kcal/day)** | 1416 (1153, 1649) | 95.0 | 17 | 67 | 16 | 1369 (1144, 1654) | 41.9 | 19 | 72 | 9 |
| **Muller (kcal/day)** | 1602 (1355, 1760) | 84.2 | 34 | 44 | 22 | 1585 (1340, 1771) | 12.1 | 36 | 47 | 17 |
| **Huang (kcal/day)** | 1550 (1285, 1752) | 83.9 | 30 | 50 | 20 | 1535 (1264, 1756) | 17.8 | 30 | 56 | 14 |
| **Wang (kcal/day)** | 1516 (1297, 1723) | 86.9 | 29 | 53 | 18 | 1498 (1291, 1726) | 28.3 | 29 | 56 | 15 |
| **Cunningham (kcal/day)** | 1484 (1264, 1692) | 90.6 | 24 | 57 | 19 | 1466 (1258, 1695) | 31.4 | 25 | 62 | 13 |
| **Johnstone (kcal/day)** | 1551 (1354, 1762) | 81.7 | 29 | 50 | 21 | 1527 (1340, 1769) | 14.4 | 35 | 49 | 16 |

BW: Body weight, REE: Resting Energy Expenditure, RMSE: Root mean square prediction error.

Data are presented as means (standard deviation) or median (and 25^th^ – 75^th^ percentiles).

^*^Predicted REE is considered accurate if it is within ±10% of measured REE.

Adjusted BW: (Current BW + Ideal BW)/2 for obese patients (BMI ≥ 30 kg/m^2^), current BW for underweight, normal-weight and overweight patients. Ideal BW is corresponded to BMI 25 kg/m^2^.

**Supplementary Table 3. Bland-Altman analyses for the agreement between measured REE from indirect calorimetry and REE from predictive equations in 186 patients with Crohn’s disease according to disease activity.**

|  | **Active disease**  **(N=70)** | | | | **Remission**  **(N=116)** | | | |
| --- | --- | --- | --- | --- | --- | --- | --- | --- |
|  | **Bias (kcal/day)*** | **Bias (%)*** | **Limits of agreement (mean ±2SD) (kcal/day)** | **95% confidence interval for the bias (kcal/day)** | **Bias (kcal/day)*** | **Bias (%)*** | **Limits of agreement (mean ±2SD)**  **(kcal/day)** | **95% confidence interval for the bias (kcal/day)** |
| **Predictive equations without body composition parameters** | | | | | | | | |
| **Schofield with current BW** | -36 | 2.48 | -729 to 658 | -120 to -49 | -100 | -2.30 | -757 to 558 | -162 to -38 |
| **Schofield with adjusted BW** | -90 | -0.55 | -762 to 583 | -171 to -8 | -131 | -4.0 | -798 to 535 | -194 to -69 |
| **FAO/WHO/UNU with current BW** | -45 | 1.84 | -738 to 647 | -130 to 39 | -105 | -2.67 | -766 to 555 | -167 to -43 |
| **FAO/WHO/UNU with adjusted BW** | -99 | -1.17 | -773 to 575 | -181 to -17 | -137 | -4.36 | -804 to 533 | -199 to -74 |
| **Harris-Benedict with current BW** | 27 | 6.07 | -685 to 738 | -60 to 113 | -32 | 1.66 | -649 to 586 | -90 to 26 |
| **Harris-Benedict with adjusted BW** | -35 | -2.57 | -708 to 637 | -117 to 47 | -67 | -0.24 | -698 to 565 | -126 to -7 |
| **Mifflin with current BW** | -146 | -4.45 | -835 to 542 | -230 to -62 | -199 | -8.56 | -805 to 408 | -256 to -142 |
| **Mifflin with adjusted BW** | -195 | -7.28 | -872 to 481 | -278 to -113 | -228 | -10.16 | -855 to 399 | -287 to -167 |
| **Owen with current BW** | -157 | -4.54 | -881 to 566 | -245 to -69 | -225 | -9.55 | -900 to 451 | -288 to -161 |
| **Owen with adjusted BW** | -201 | -7.02 | -912 to 510 | -288 to -115 | -249 | -10.9 | -940 to 441 | -314 to -185 |
| **Muller with current BW** | -72 | 0.42 | -791 to 647 | -159 to -16 | -141 | -4.90 | -787 to 504 | -202 to -81 |
| **Muller with adjusted BW** | -131 | -2.96 | -824 to 563 | -215 to -46 | -177 | -6.82 | -839 to 485 | -239 to -115 |
| **Marra with current BW** | -12 | 4.20 | -719 to 696 | -98 to -74 | -62 | 0.17 | -686 to 562 | -121 to -4 |
| **Marra with adjusted BW** | -65 | 1.14 | -752 to 622 | -148 to -19 | -94 | -1.56 | -735 to 546 | -154 to -34 |
| **Predictive equations without body composition parameters** | | | | | | | | |
| **Mifflin-St. Jeor** | -269 | -11.6 | -983 to 446 | -357 to -180 | -298 | -14.1 | -959 to 360 | -360 to -236 |
| **Owen** | -281 | -12.8 | -1004 to 442 | -371 to -191 | -320 | -15.9 | -979 to 339 | -382 to -258 |
| **Muller** | -95 | -0.81 | -818 to 628 | -185 to -5 | -161 | -5.78 | -805 to 484 | -221 to -100 |
| **Huang** | -141 | -3.93 | -863 to 562 | -231 to -51 | -207 | -8.96 | -850 to 436 | -268 to -147 |
| **Wang** | -181 | -6.25 | -895 to 534 | -270 to -92 | -211 | -8.90 | -863 to 441 | -273 to -150 |
| **Cunningham** | -213 | -8.26 | -927 to 502 | -302 to -124 | -243 | -10.9 | -895 to 409 | -304 to -182 |
| **Johnstone** | -130 | -3.34 | -848 to 589 | -219 to -40 | -170 | -6.72 | -793 to 454 | -229 to -111 |

BW: Body weight, REE: Resting Energy Expenditure.

*Bias is defined as the mean difference between predicted REE and measured REE.

Adjusted BW: (Current BW + Ideal BW)/2 for obese patients (BMI ≥ 30 kg/m^2^), current BW for underweight, normal-weight and overweight patients. Ideal BW is corresponded to BMI 25 kg/m^2^.

**Supplementary Figure 1. Comparison of measured REE and REE from predictive equations in 186 patients with Crohn’s disease using current body weight for all patients for equations without body composition analysis parameters (A), using adjusted body weight* only for obese patients for equations without body composition analysis parameters (B) and for equations with body composition analysis parameters (C).**

1. **For current body weight**


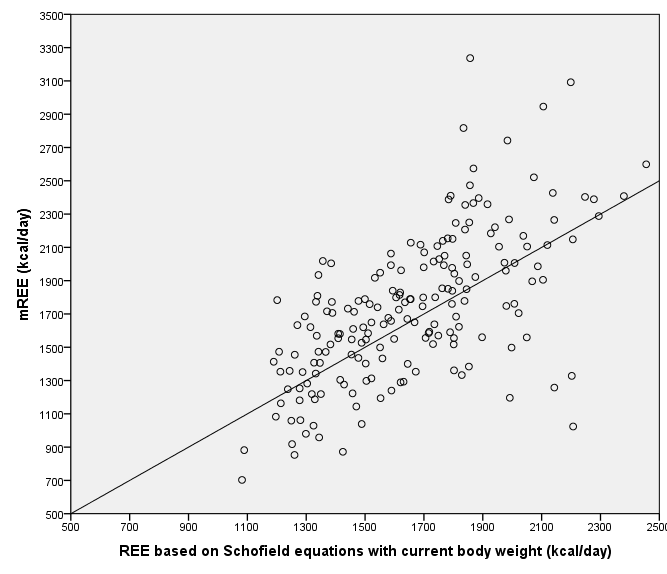

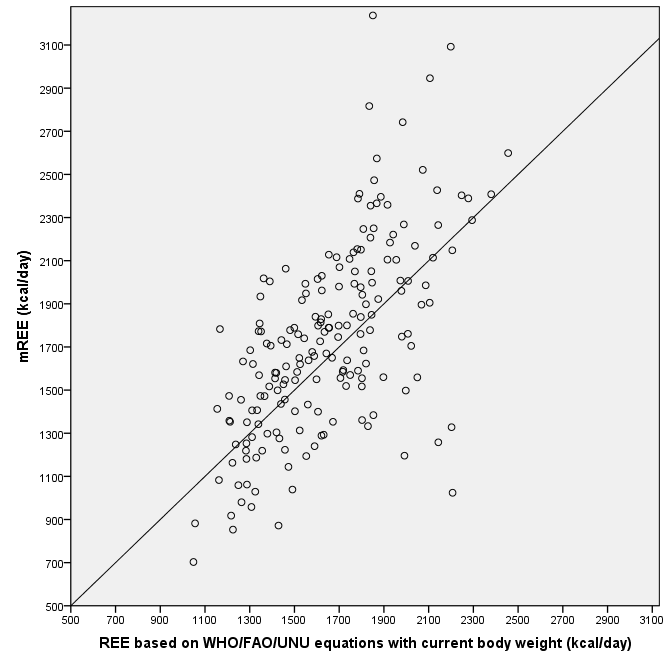

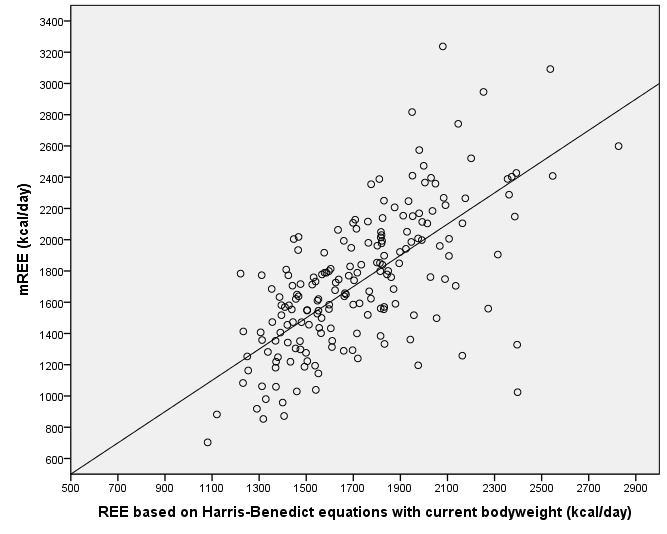

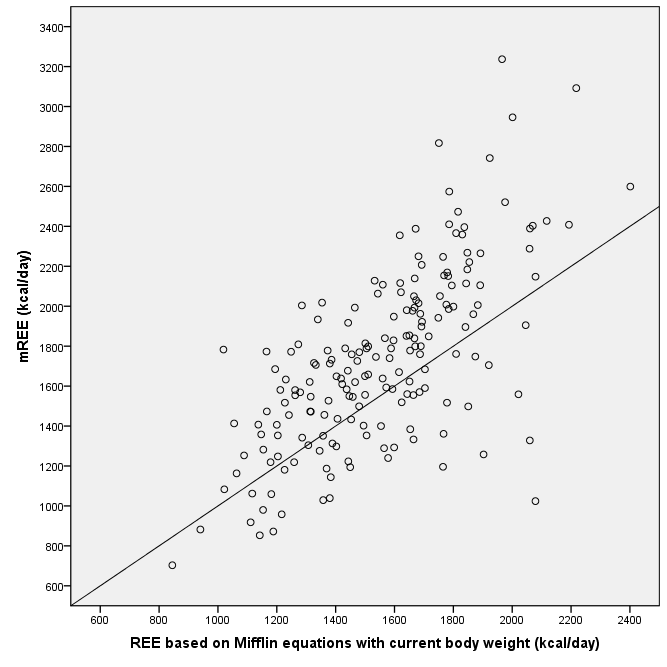

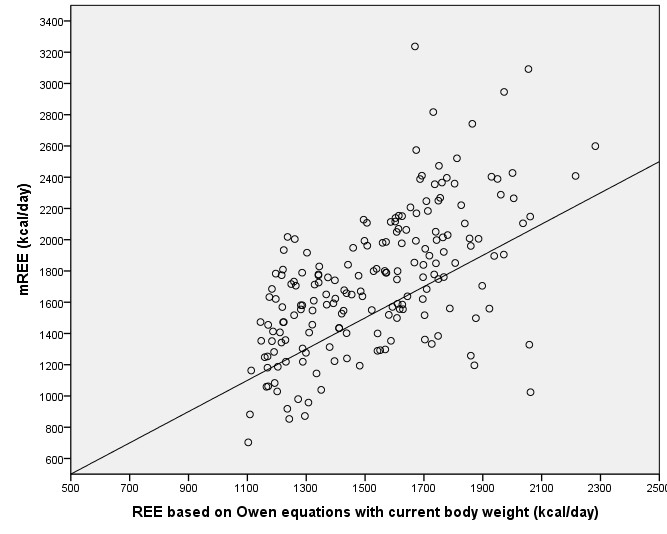

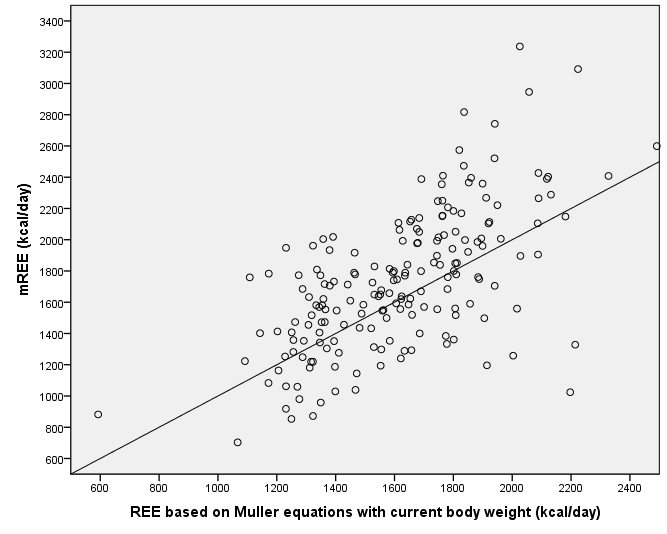

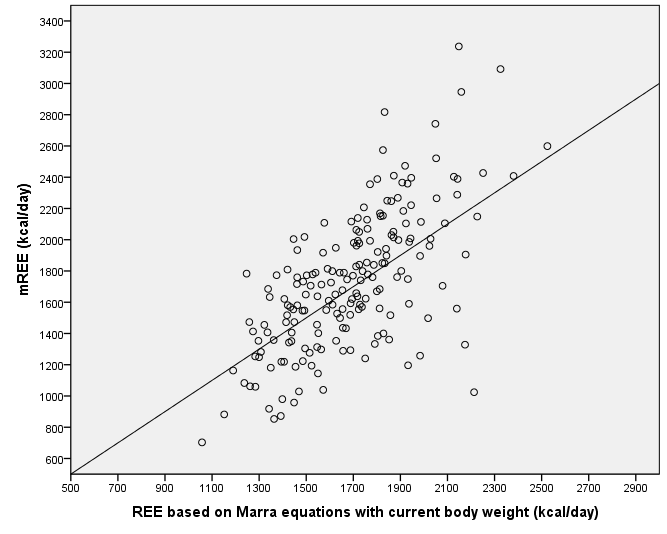


1. **For adjusted body weight***


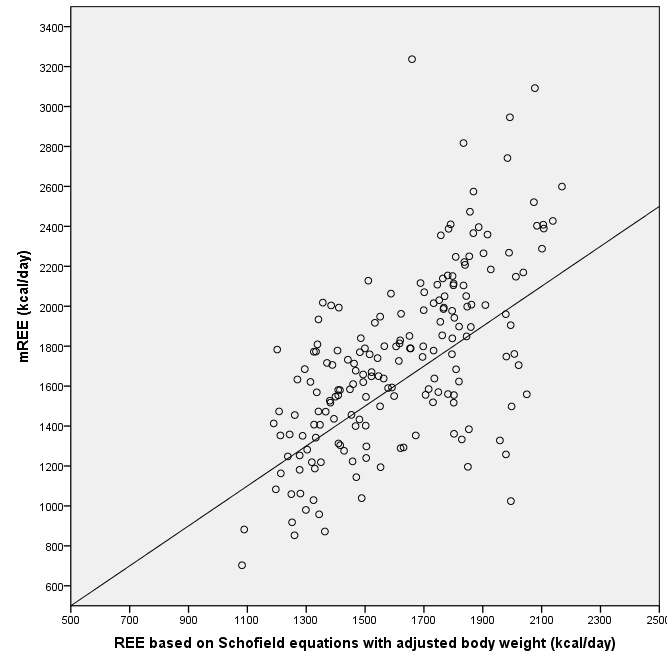

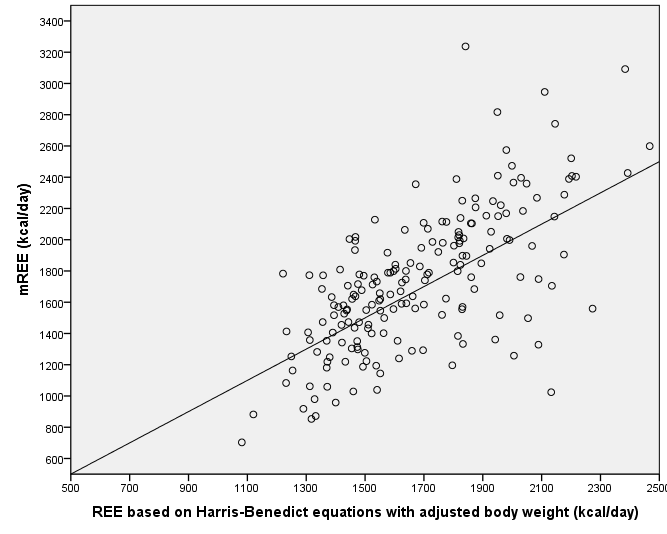

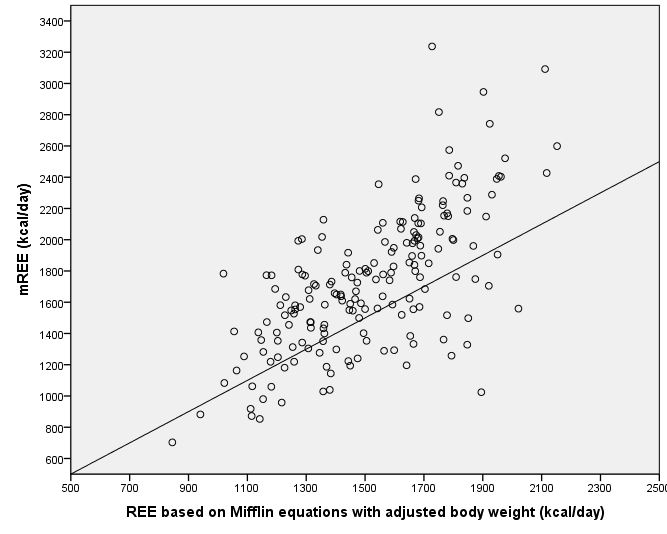

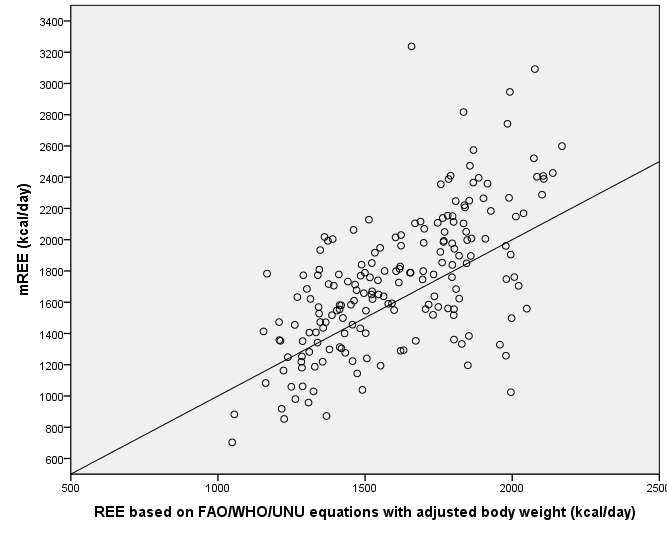

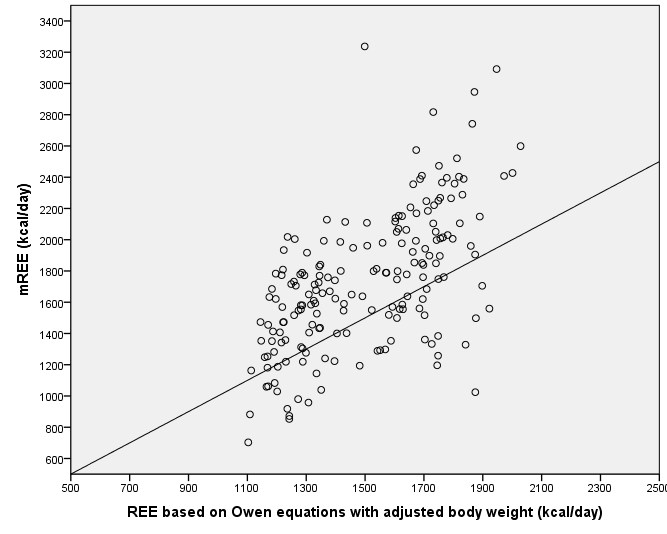

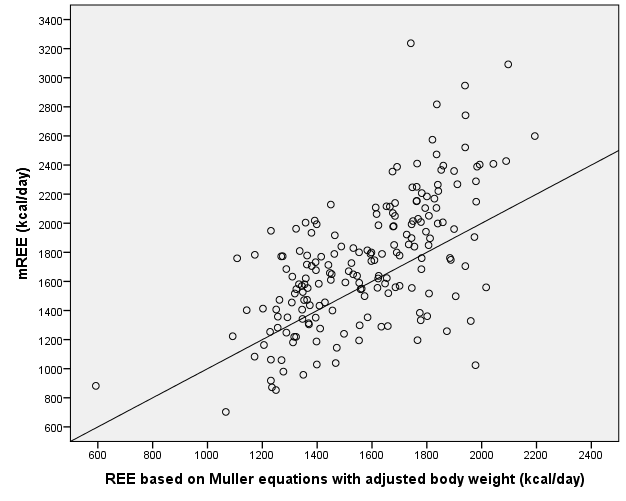

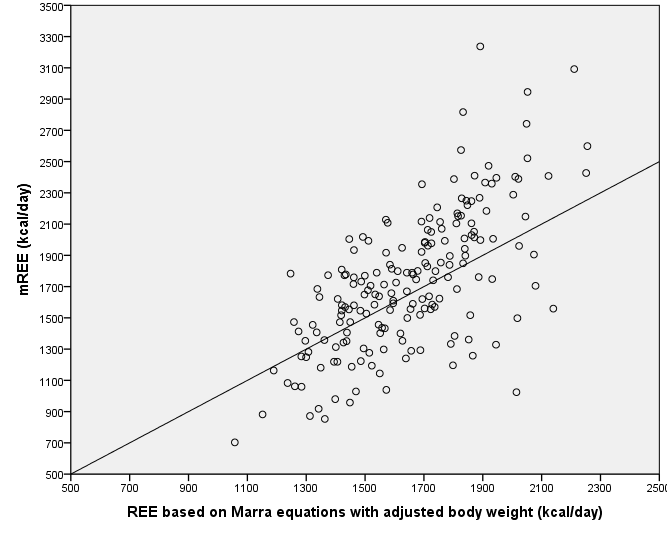


1. **For equations with body composition analysis parameters**


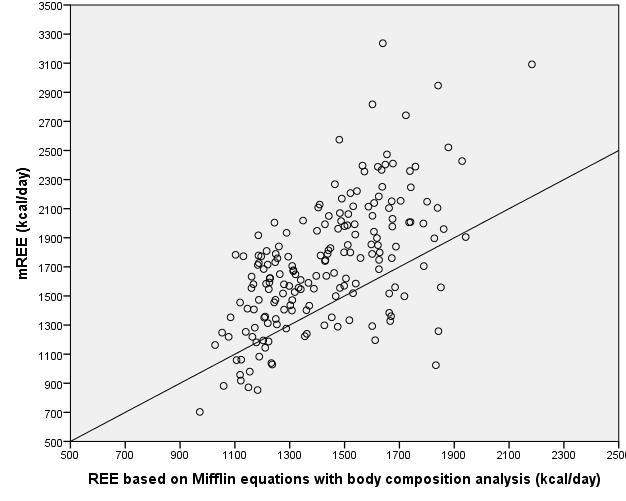

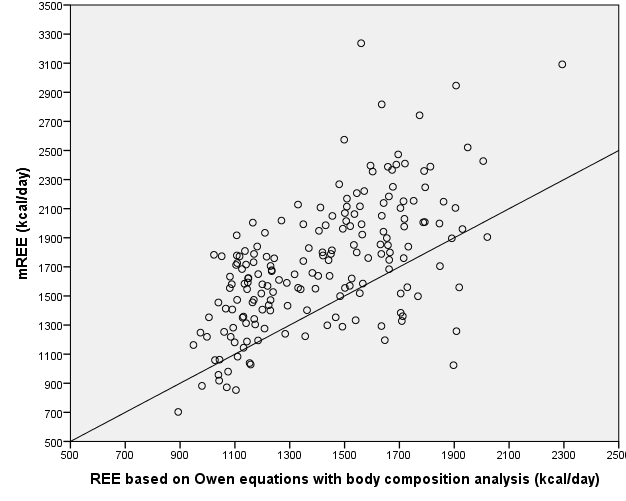

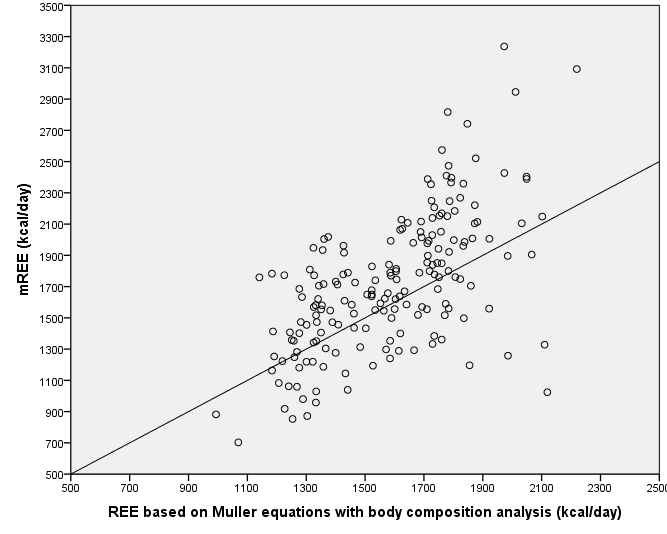

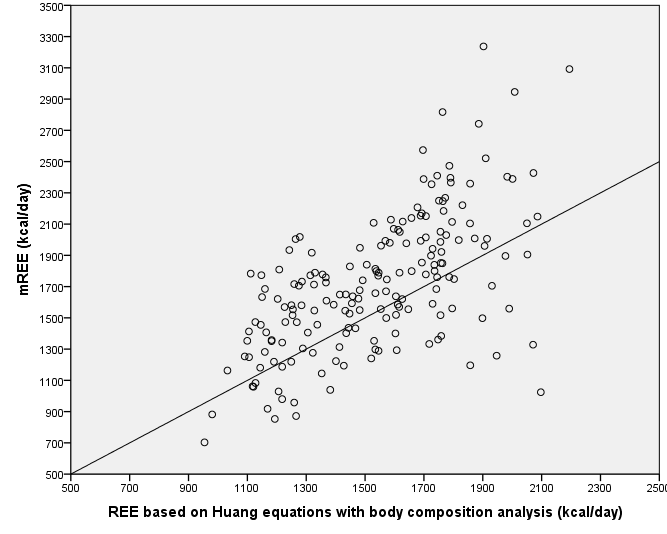

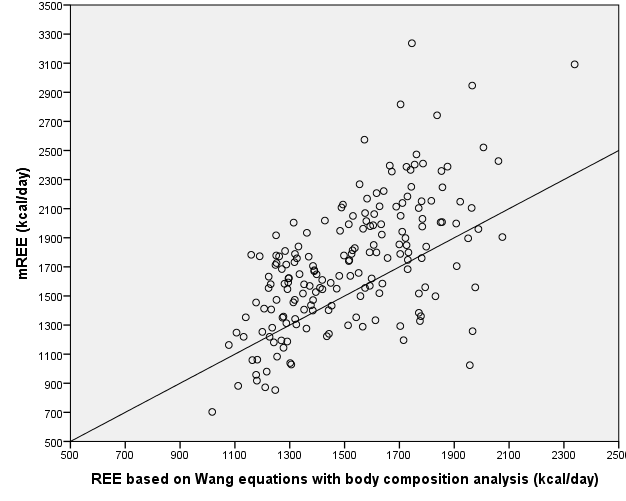

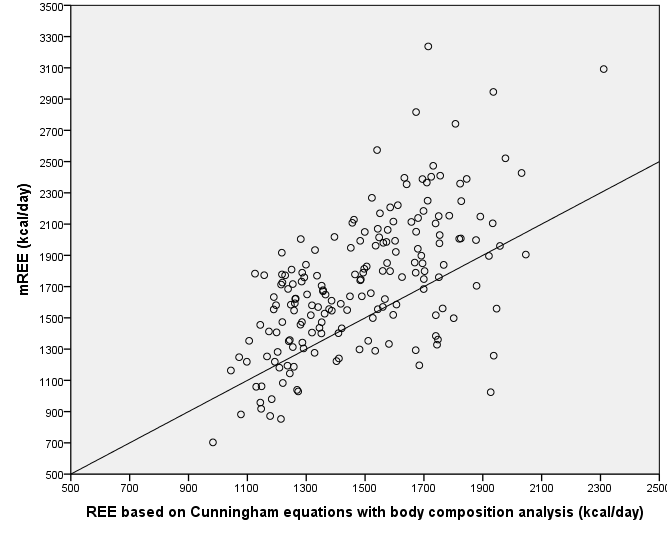

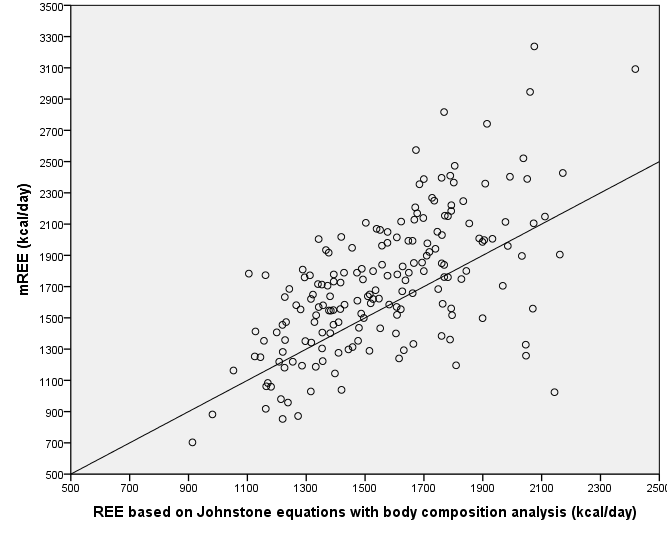


REE: Resting energy expenditure, mREE: measured resting energy expenditure for indirect calorimetry, pREE: predicted resting energy expenditure from predictive equations

*Adjusted body weight: (Current body weight + Ideal body weight)/2 for obese patients (BMI ≥ 30 kg/m^2^), current body weight for underweight, normal-weight and overweight patients. Ideal body weight is corresponded to BMI 25 kg/m^2^.

**Supplementary Figure 2. Bland-Altman analyses charts** **for the agreement between measured REE and REE from predictive equations in 186 patients with Crohn’s disease using current body weight for all patients for equations without body composition analysis parameters (A), using adjusted body weight* only for obese patients for equations without body composition analysis parameters (B) and for equations with body composition analysis parameters (C).**

1. **For current body weight for all patients for equations without body composition analysis parameters**


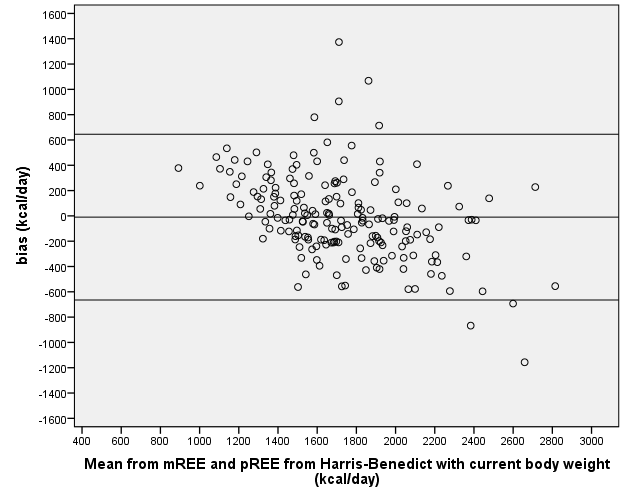

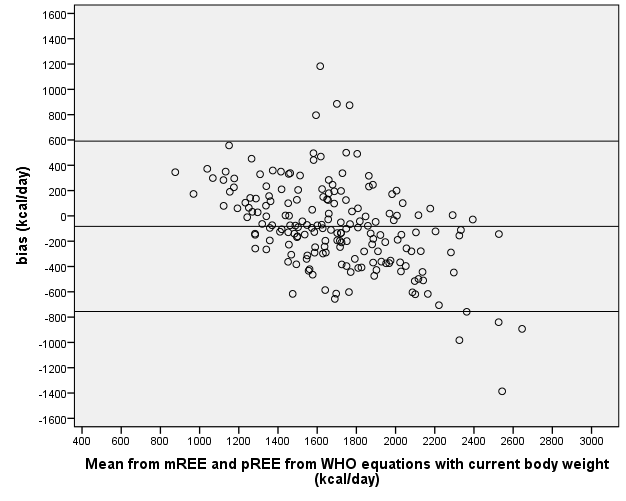

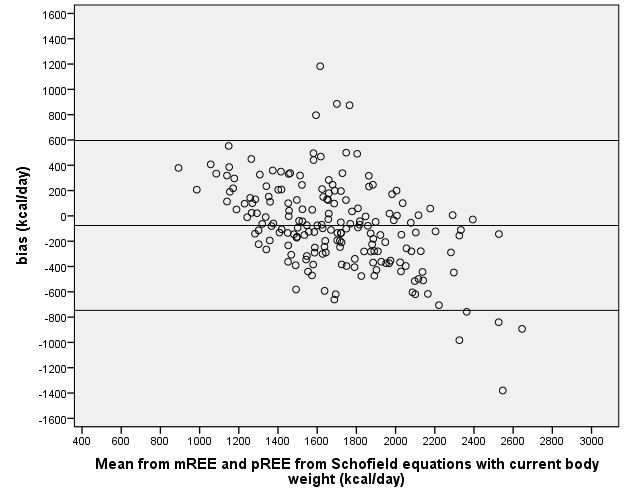

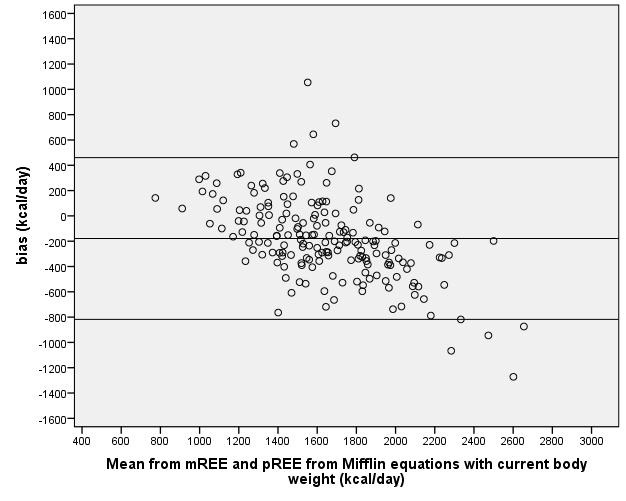

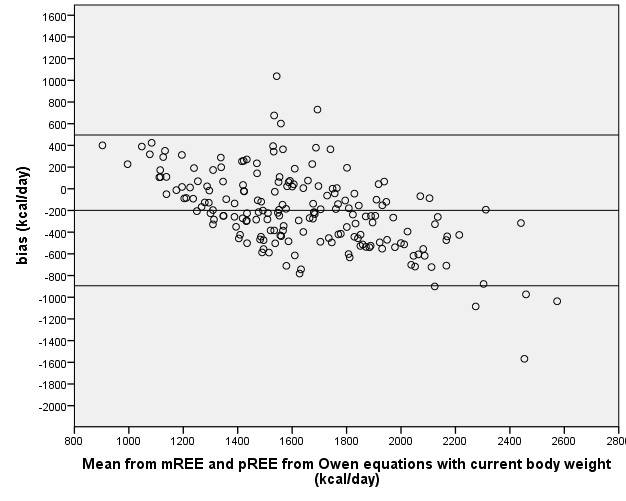

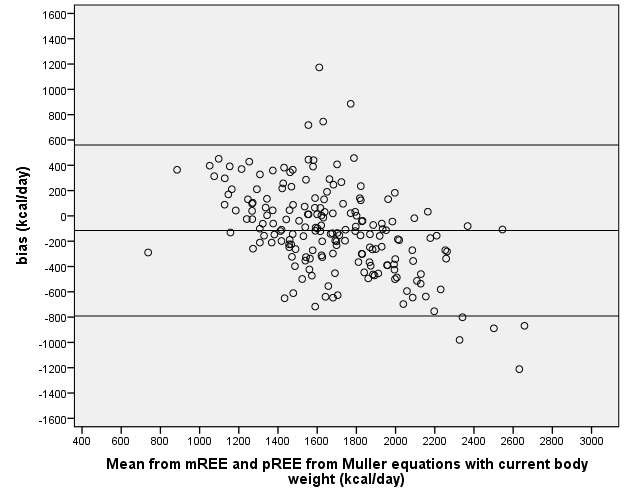

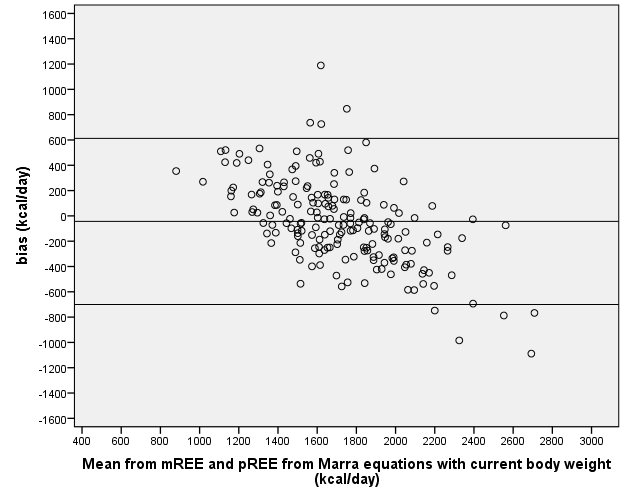


1. **For adjusted body weight* only for obese patients for equations without body composition analysis parameters.**


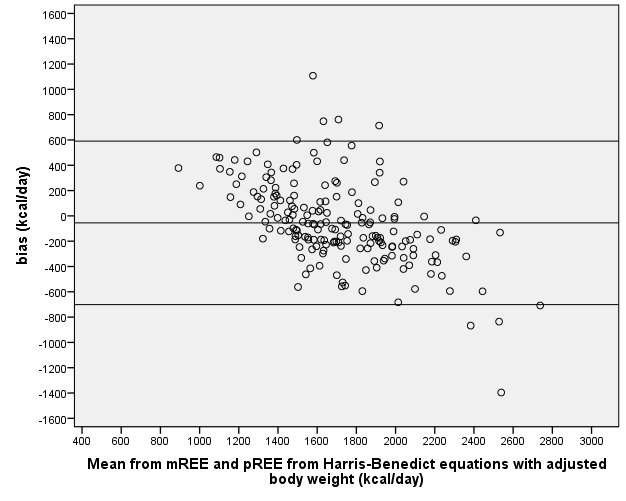

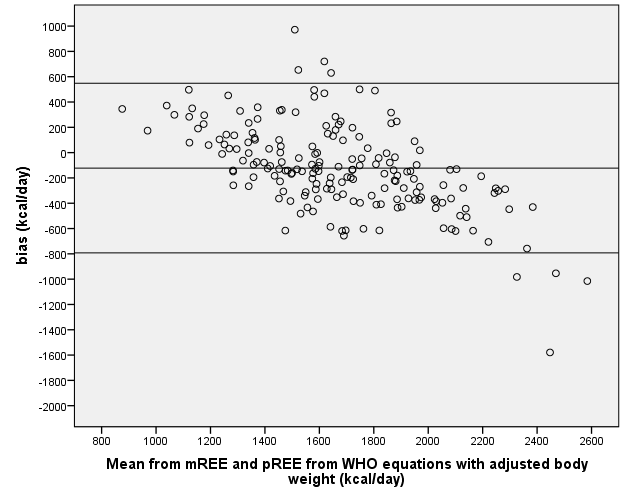

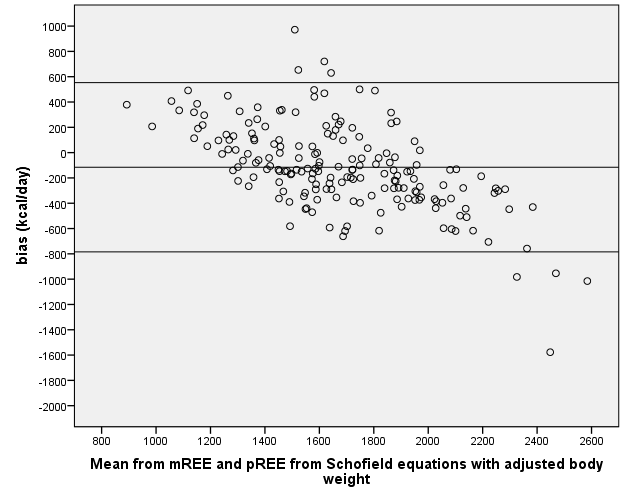

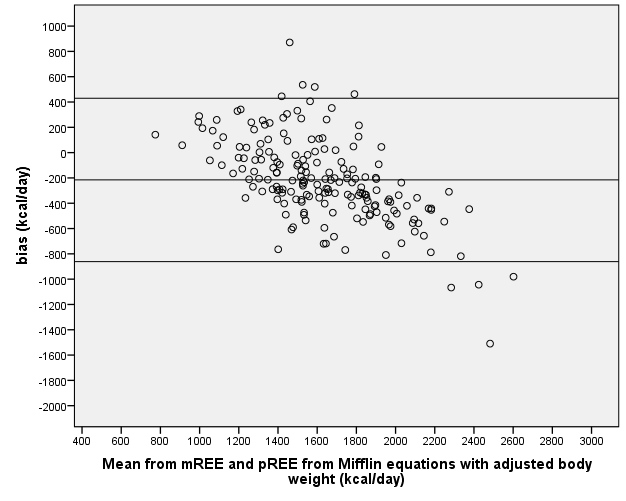

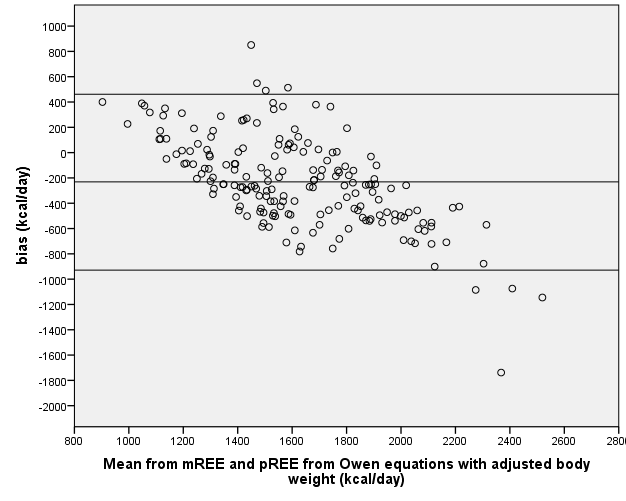

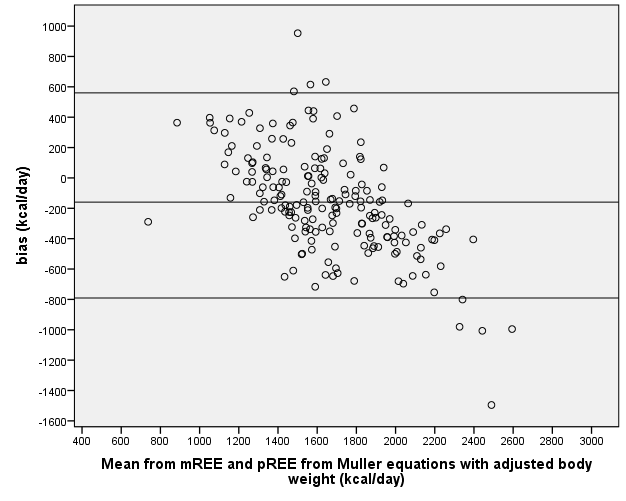

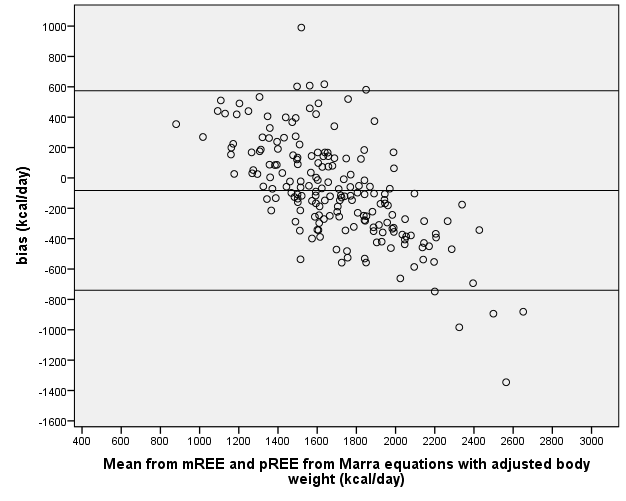


1. **For equations with body composition analysis parameters**


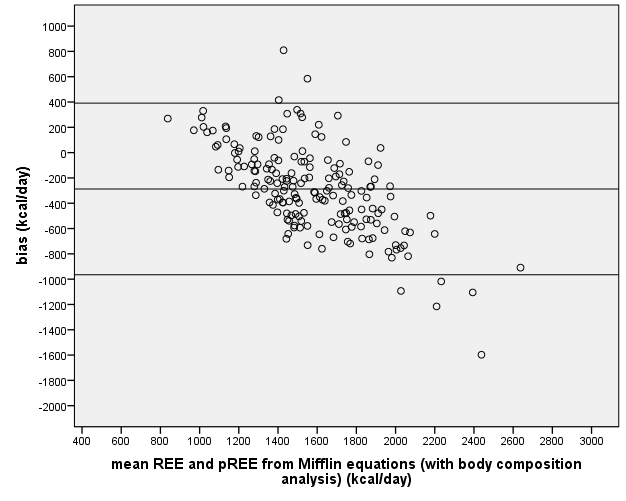

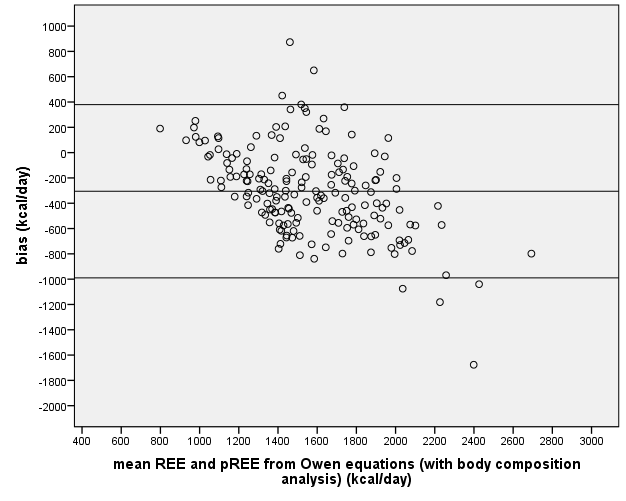

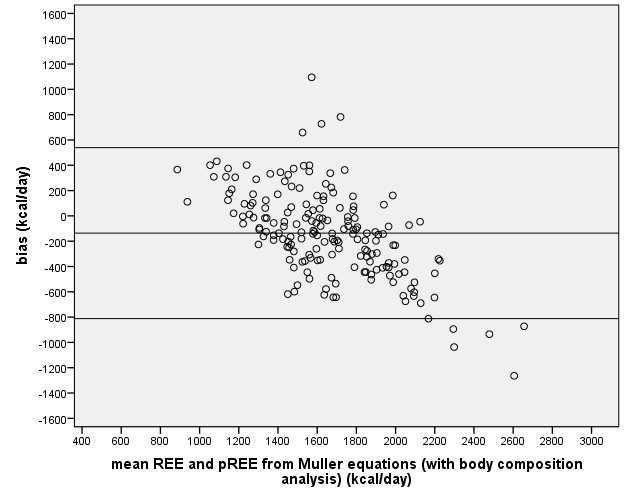

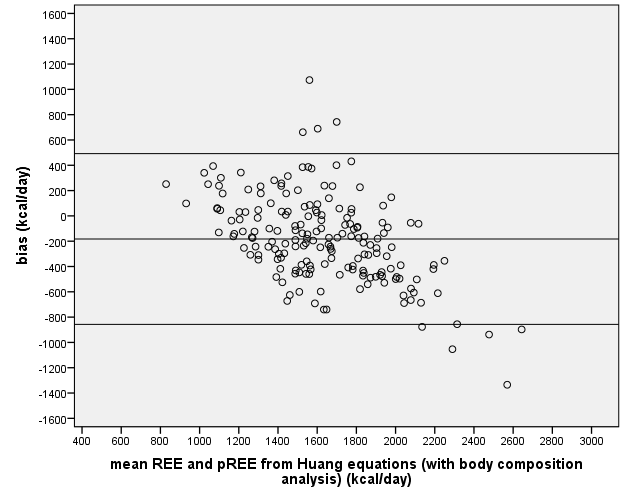

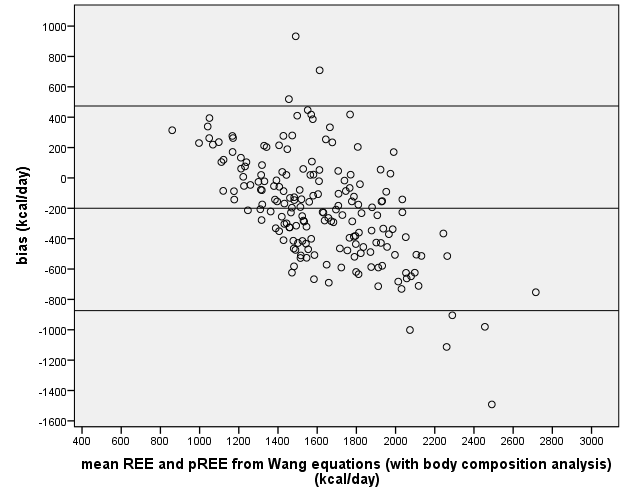

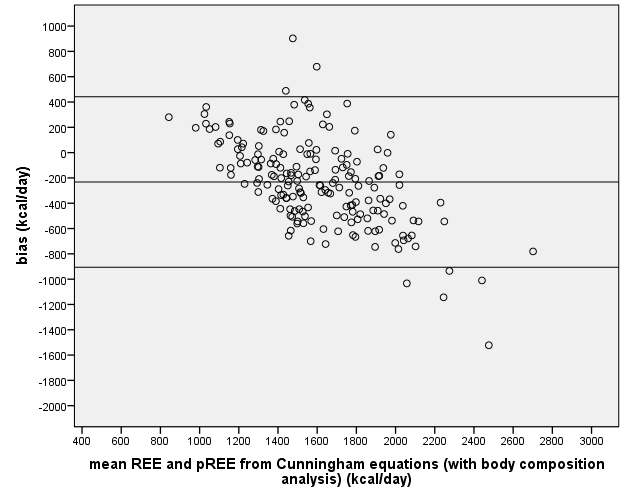

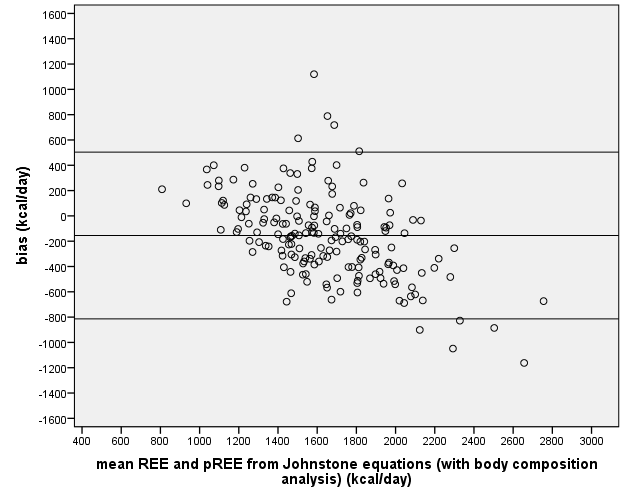


REE: Resting energy expenditure, mREE: measured resting energy expenditure for indirect calorimetry, pREE: predicted resting energy expenditure from predictive equations

Bias is calculated as pREE-mREE.

*Adjusted body weight: (Current body weight + Ideal body weight)/2 for obese patients (BMI ≥ 30 kg/m^2^), current body weight for underweight, normal-weight and overweight patients. Ideal body weight is corresponded to BMI 25 kg/m^2^.

**Supplementary Figure 3. Accuracy between measured (mREE) and predicted REE (pREE) using current body weight for 186 patients with Crohn’s disease according to Body Mass Index (BMI) categories (<18.5 kg/m^2^, 18.5-24.9 kg/m^2^, 25-26.9 kg/m^2^, 27-29.9 kg/m^2^ and ≥30 kg/m^2^) for all equations.**


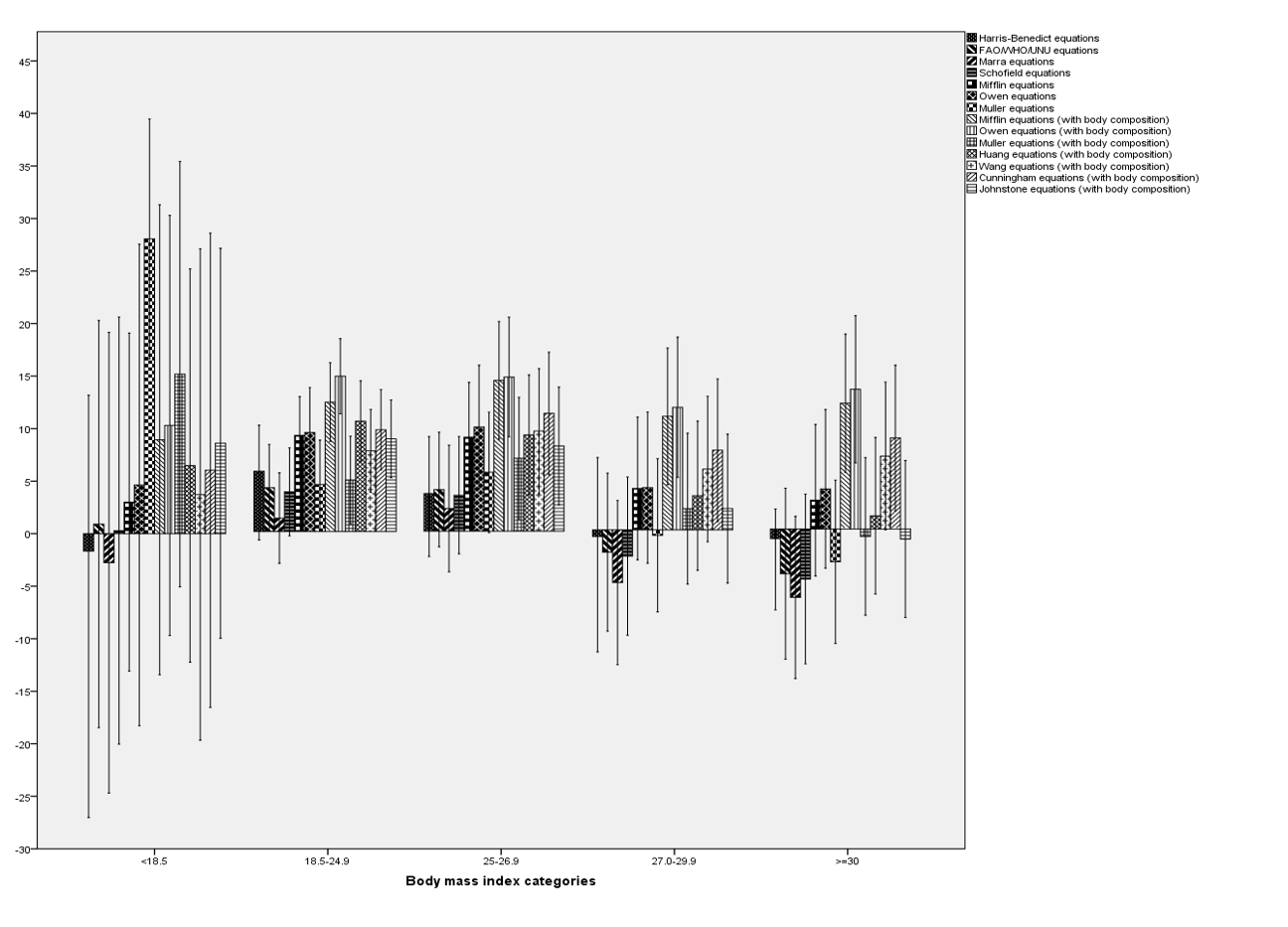


Accuracy was calculated as: (pREE-mREE/mREE) * 100 and is presented as mean percentage with 95% confidence intervals. All REE equations without body composition parameters were calculated with current body weight.

*Abbreviations:* REE: Resting energy expenditure.
